# Transcriptional and metabolic modeling analyses of developing *Aspergillus fumigatus* biofilms reveal metabolic shifts required for biofilm maturation

**DOI:** 10.1128/msphere.00752-25

**Published:** 2025-11-28

**Authors:** Charles Puerner, Kaesi A. Morelli, Joshua D. Kerkaert, Jane T. Jones, Katherine G. Quinn, Nathan DeMichaelis, Sandeep Vellanki, Chen Liao, Robert A. Cramer

**Affiliations:** 1 Department of Microbiology and Immunology, Geisel School of Medicine, Dartmouth 12285, Hanover, New Hampshire, USA; Shenzhen Institute of Synthetic Biology, Shenzhen, China

**Keywords:** *Aspergillus fumigatus*, hypoxia, biofilm, biofilm maintenance, fermentation, metabolic flux analysis

## Abstract

*Aspergillus fumigatus* is a filamentous fungus found in compost and soil that can cause invasive and/or chronic disease in humans. Diagnosis and treatment of aspergillosis often occur when *A. fumigatus* has formed dense networks of hyphae within the lung. These hyphal networks are multicellular, encased in an extracellular matrix, and have reduced susceptibility to contemporary antifungal drugs, similar to bacterial biofilms. A model of these dense hyphal networks observed *in vivo* can be recapitulated *in vitro* using a static, submerged biofilm culture. The mechanisms underlying filamentous fungal cell physiology at different stages of biofilm development remain to be defined. Here, we utilized RNA sequencing, *in silico* metabolic modeling, and molecular genetics approaches to identify *A. fumigatus* genes and metabolic pathways critical for biofilm development. These analyses revealed that ethanol and butanediol fermentation pathways are important for the development of a mature *A. fumigatus* biofilm. Correspondingly, a predicted transcription factor (*silG*) was observed to be required for mature biofilm development. Taken together, these data define key genes and metabolic pathways critical for *A. fumigatus* biofilm development.

**IMPORTANCE:**

*Aspergillus fumigatus* is the most common etiological agent of a collection of diseases termed aspergillosis. Chronic and invasive manifestations of aspergillosis are highlighted by the development of biofilm-like structures on and in tissue. These biofilm structures are resistant to contemporary antifungal drugs, even for strains that are susceptible by standard antimicrobial susceptibility testing methods. Consequently, understanding the mechanisms by which *A. fumigatus* induces, develops, and maintains biofilms to evade antifungal therapies is expected to illuminate biofilm-specific therapeutic targets. Here, we identify genes involved in fungal fermentation and regulation of transcription as important mediators of *A. fumigatus* biofilm development.

## INTRODUCTION

*Aspergillus fumigatus* is a ubiquitous filamentous fungus that causes a broad spectrum of acute and chronic diseases collectively termed aspergillosis. Mortality of aspergillosis in all its manifestations remains high, even with antifungal treatment, and strains that respond to antifungal drugs *in vitro* frequently fail to respond to corresponding treatment *in vivo* (1). As a diagnosis of aspergillosis can be challenging, antifungal treatment typically does not occur until the fungus has established mature, dense hyphal networks on or within host tissue. These hyphal networks at sites of infection have hallmark characteristics of a microbial biofilm, including multicellularity, extracellular polysaccharide secretion, surface adherence, limited oxygen availability, and drug resistance (2–7).

Importantly, the low-oxygen environments that arise during biofilm maturation are in part responsible for the observed biofilm antifungal resistance (3). An additional mechanism of biofilm drug resistance is also mediated by the presence of extracellular DNA in the extracellular matrix (8). However, the mechanisms through which *A. fumigatus* biofilms fully develop and maintain their antifungal drug resistance remain ill-defined (9, 10). Identifying biofilm-specific antifungal drug resistance mechanisms is expected to help develop new therapeutic targets to increase antifungal efficacy *in vivo* when robust biofilm-mediated infections are established.

To help fill the knowledge gap on *A. fumigatus* biofilm development mechanisms, we utilized a submerged biofilm culture model to identify genes expressed at different timepoints of *A. fumigatus* biofilm development. The transcriptomics data suggest a shift from oxidative phosphorylation-mediated energy generation toward a more fermentative metabolism as the biofilm matures. Development of an *in silico* metabolic flux model supported the interpretation of the transcriptomics data and further predicted key metabolic rewiring that occurs during biofilm development. In support of the emerging metabolic model, loss of genes required for ethanol and butanediol fermentation reduced the biomass and biovolume of mature biofilms. Additionally, we identified a previously uncharacterized predicted transcription factor whose transcript levels increase throughout biofilm development and are required for mature biofilm development. These data provide insight into the genes and metabolic pathways important for *A. fumigatus* submerged biofilm development and reveal a previously unidentified transcriptional regulator as a mediator of mature biofilm development.

## RESULTS

### Biofilm developmental stages are transcriptionally unique

**Fig 1:**
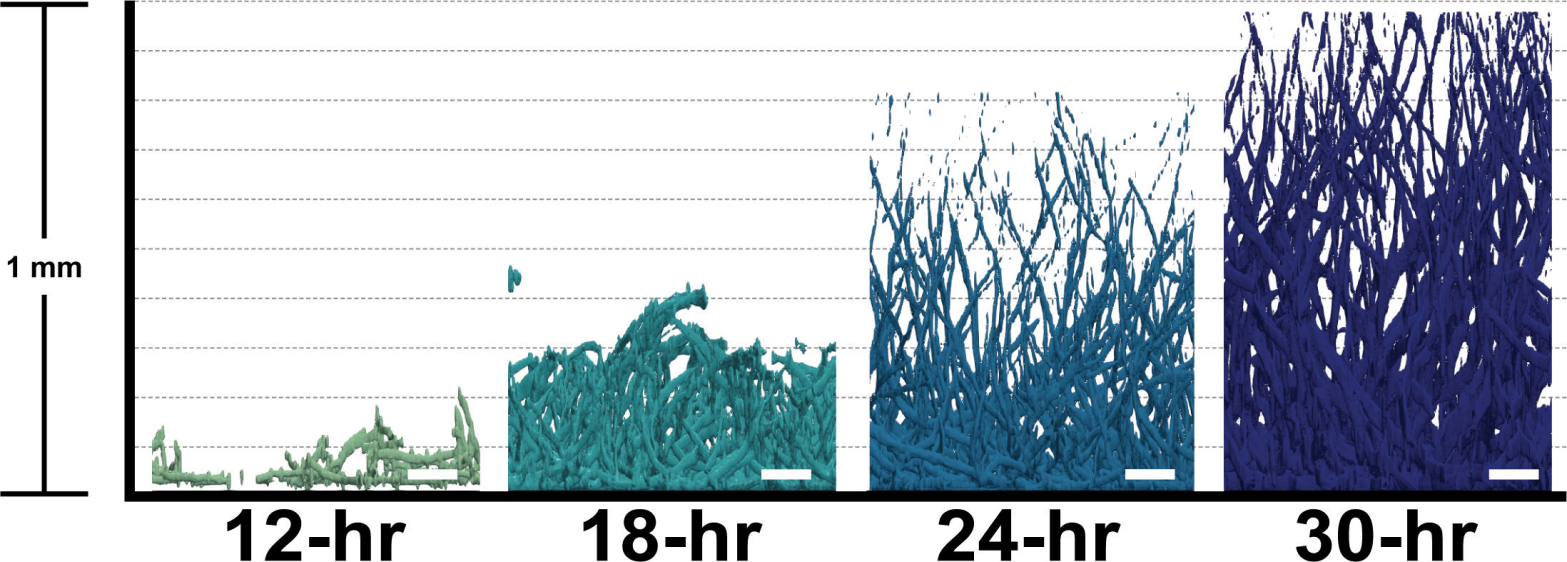
Morphology of biofilm development. Z-stack images acquired at 12-, 18-, 24-, and 30-hour timepoints during development of the *A. fumigatus* biofilm. Biofilms were stained with calcofluor white prior to imaging. Fluorescence images were rendered using BiofilmQ and Paraview software. The scale bar is 100 µm.

To identify genes and pathways that are important during distinct stages of *A. fumigatus* submerged biofilm development, we utilized an RNA-Seq approach. The *A. fumigatus* laboratory strain, CEA10 (also known as Fungal Genetics Stock Center strain A1163), was utilized to generate submerged biofilms in glucose minimal medium with nitrate as the nitrogen source for 12, 18, 24, and 30 hours of biofilm development (Fig. 1). These timepoints in this model were chosen based on our previous analyses of biofilm architecture and antifungal drug susceptibility (3). Bulk RNA was extracted from replicate biofilms at each timepoint and analyzed by single-end Illumina RNA sequencing. We acquired a mean of 10.1 M reads per sample (SD = 5.5 M) and had a mean mapping rate of 81.2% (SD = 3.6%) to the A1163 reference genome (11).

**Fig 2:**
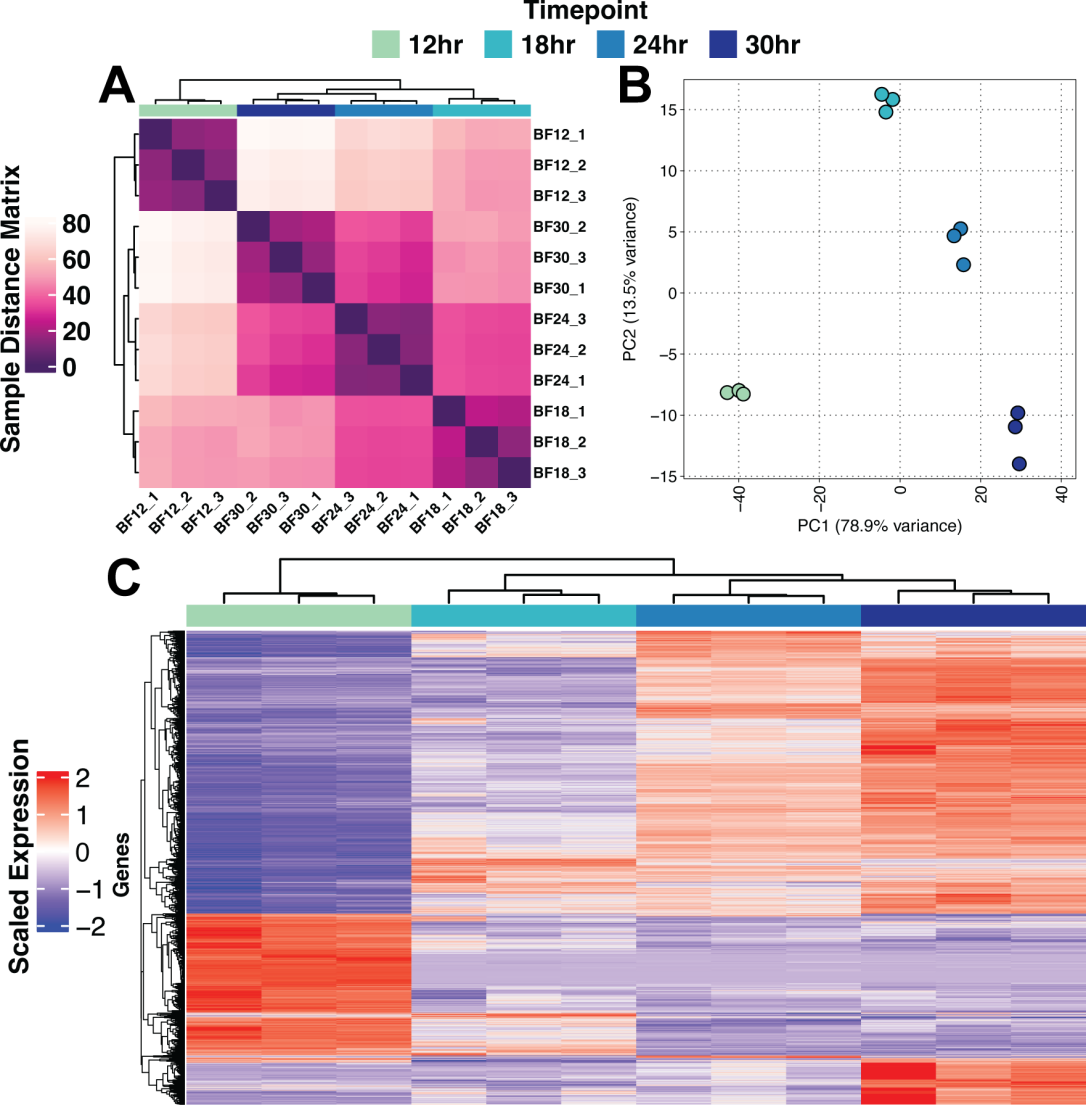
Exploratory analysis of the *A. fumigatus* biofilm transcriptional landscape over time. (**A**) Euclidean distance matrix representing distance relationships between samples. BF indicates biofilm with timepoint number and replicate number following. (**B**) PC analysis shows separation of timepoints in the first PC. (**C**) Hierarchical clustering of the top 2,000 most variable genes shows progression of biofilm development, with the 12-hour timepoint showing a unique state compared to the remaining timepoints. Scaled vst normalized values are shown in the heatmap. The heatmap shows likely timepoint-specific gene expression patterns.

Exploratory analysis revealed that each biofilm stage is transcriptionally unique. Global Euclidean clustering of all samples shows samples within each timepoint are closer than samples from other timepoints (Fig. 2A). Principal component (PC) analysis of the top 2,000 most variable transcripts further confirms the intra-timepoint clustering and a clear inter-timepoint separation (Fig. 2B). The top 2,000 most variable genes were used in this analysis as this captured the majority of the variation in the data set (Fig. S1). PC 1 (78.9% of variance) is explained by a clear stepwise separation of biofilm development along this PC, while PC2 (13.5% of variance) is seemingly explained by developing biofilm versus immature and mature biofilms. From both exploratory analyses, we determined that 12-hour biofilm samples are transcriptionally distinct from the three later biofilm stages. A hierarchical clustering analysis of transcript abundance dynamics of the top 2,000 most variable transcripts revealed potential clusters of genes important for each stage of development (Fig. 2C). For example, clusters of genes with transcripts that are increased at 18 hours when compared to 12 hours but subsequently reduced in abundance at 24 hours compared to 12 hours suggest that these transcripts are specifically important for the 18 hours of stage of biofilm development (Table S1).

### Investigation of timepoint-specific expression patterns

**Fig 3:**
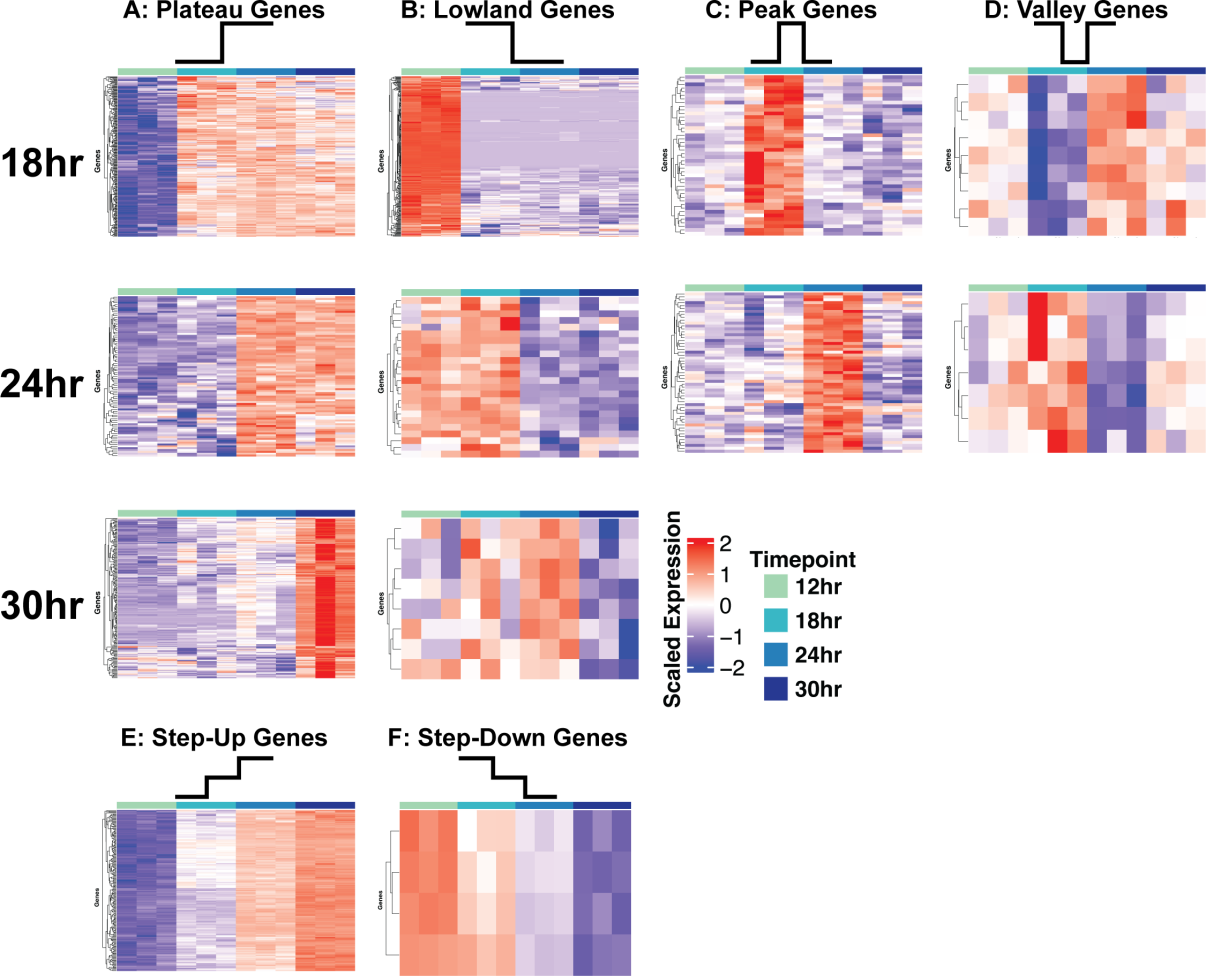
Identification of dynamic biofilm gene expression patterns. Using differential expression data (log2FC and adjusted *P*-value), dynamic gene expression patterns were identified for each timepoint. (**A**) Plateau genes increase in expression at a given timepoint and remain increased during development. (**B**) Lowland genes decrease in expression at a given timepoint and remain lowly expressed during the remainder of development. (**C**) Peak genes increase in expression specifically at 18- or 24-hour timepoints. (**D**) Valley genes decrease specifically at 18- or 24-hour timepoints. (**E**) Step-up genes increase incrementally throughout biofilm development. (**F**) Step-down genes decrease incrementally throughout biofilm development. Scaled CPM values are shown.

We utilized a pairwise differential expression analysis comparing all the possible combinations of pairwise comparisons between the four timepoints (six comparisons) (Table 1; Table S2). Through the exploratory and differential expression analyses, we observed clusters of transcripts that are likely regulated at specific timepoints. To explore this observation further, an analysis to identify genes that have transcript levels regulated at specific timepoints was conducted using six criteria: (i) significantly increased in transcript abundance and remain increased for all subsequent timepoints (plateau), (ii) significantly decreased in abundance and remain low for all subsequent timepoints (lowland), (iii) significantly increased in abundance at a specific timepoint (peak), (iv) significantly decreased in abundance at a specific timepoint (valley), and (v) transcripts which progressively increase in abundance over the time course (step-up) and (vi) transcripts which progressively decrease in abundance over the time course (step-down). The analysis was conducted using log_2_ fold-change and an adjusted *P*-value cutoff of significance, with criteria used for each timepoint outlined in Table 2. From this analysis, we identified clusters of plateau, lowland, peak, and valley transcripts for each timepoint, as well as step-up and step-down transcripts across the timepoints (Fig. 3; Table S3). We utilized a functional enrichment analysis using the FunCat categories from the FungiFun3 resource (12) (Table S4).

**TABLE 1:** Summary of differential gene expression analysis

Comparison	log2FC = 0			log2FC = 1			log2FC = 2		
	Total	Increase	Decrease	Total	Increase	Decrease	Total	Increase	Decrease
BF18hr_vs_BF12hr	4,119	2,010	2,109	705	460	245	470	271	199
BF24hr_vs_BF12hr	5,540	2,730	2,810	1,153	859	294	787	567	220
BF30hr_vs_BF12hr	5,783	2,915	2,868	1,456	1,162	294	1,062	838	224
BF24hr_vs_BF18hr	2,571	1,451	1,120	279	259	20	159	150	9
BF30hr_vs_BF18hr	3,160	1,957	1,203	687	651	36	454	439	15
BF30hr_vs_BF24hr	2,059	1,404	655	234	224	10	153	151	2

**TABLE 2:** Criteria used to identify peak, plateau, lowland, and step-up genes

		18 hours:12 hours		24 hours:18 hours		30 hours:24 hours	
Type	Timepoint	log2FC	adj. *P*-value	log2FC	adj. *P*-value	log2FC	adj. *P*-value
Plateau	18 hours	>1	<0.05	na	>0.05	na	>0.05
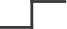	24 hours	na	>0.05	>1	<0.05	na	>0.05
	30 hours	na	>0.05	na	>0.05	>1	<0.05
Lowland	18 hours	< −1	<0.05	na	> 0.05	na	>0.05
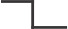	24 hours	na	>0.05	< −1	<0.05	na	>0.05
	30 hours	na	>0.05	na	>0.05	< −1	<0.05
Step-up	All	>0.5	<0.05	>0.5	<0.05	>0.5	<0.05
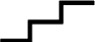							
Step-down	All	< −0.5	<0.05	< −0.5	<0.05	< −0.5	<0.05
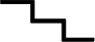							

The results of these analyses suggested that metabolic shifts take place over the course of biofilm development. For example, FunCat categories for secondary metabolism and toxins were significantly enriched in the 24- and 30-hour plateau gene lists. Due to this finding, we investigated the transcript abundances of several secondary metabolites and related pathways identified using the KEGG database (Fig. S2). We observed several biosynthetic gene clusters for secondary metabolite biosynthesis with increased transcript abundance in late stages of biofilm development (Fig. S3). Specifically, we found the following biosynthetic pathways to have increased transcripts: Fumagillin, Fumigaclavine, Fumiquinazoline, and Fumitremorgin. As these pathways appear to be turning on as the biofilms mature, it is an intriguing possibility that they are involved in biofilm maturation and/or maintenance.

### Transcripts associated with fermentative metabolism increase over time

**Fig 4:**
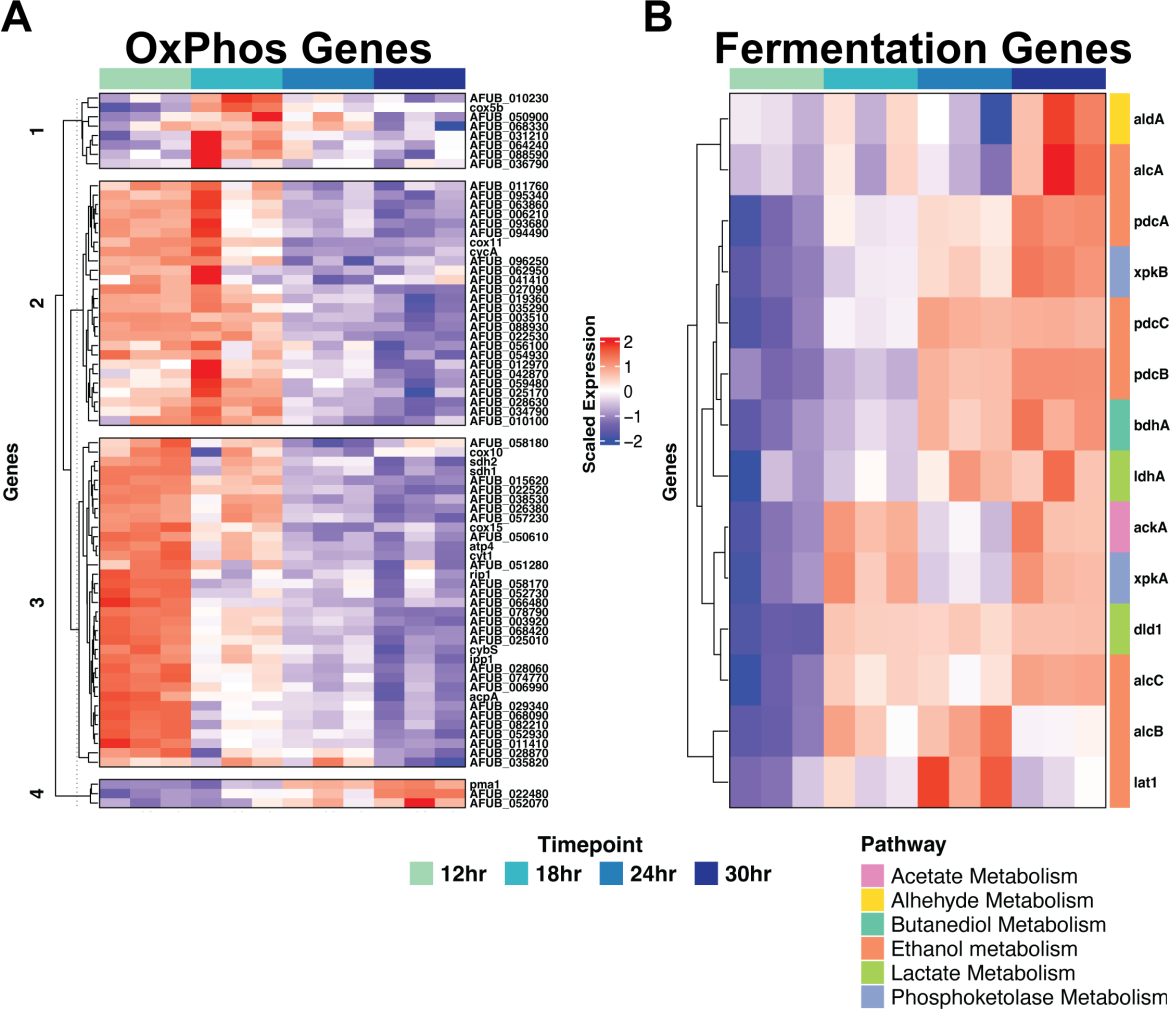
Gene expression of oxygen-dependent and -independent energy production pathways. Gene expression from genes within (**A**) oxidative phosphorylation (OxPhos) and (**B**) fermentation pathways showed a general decrease in oxidative phosphorylation genes and a general increase in fermentative genes. Fermentative pathways are indicated by the color key, and scaled CPM values are shown in the heatmaps. K-means cluster number is indicated to the left of the heatmap.

To further define the metabolic changes occurring during biofilm development, we compared transcript levels of genes associated with oxidative phosphorylation and fermentative metabolism at each stage of biofilm development. Using the KEGG database, we identified genes associated with oxidative phosphorylation to subset our data (13). In support of our hypothesis that metabolic re-wiring occurs during biofilm development, transcripts for the majority of genes associated with oxidative phosphorylation were highest at the 12-hour timepoint compared to all other timepoints (Fig. 4A). Biofilms at 18 hours had relatively high transcript abundance of genes encoding proteins predicted to function in oxidative phosphorylation, though some heterogeneity is observed at this timepoint. By the 24-hour timepoint, there is a relative reduction in the mRNA abundance of oxidative phosphorylation-related genes. Oxidative phosphorylation-associated transcripts are further reduced in 30-hour biofilms.

Conversely, comparison of transcript levels of genes encoding proteins associated with fermentative metabolism and the phosphoketolase pathway revealed an opposite transcript abundance pattern compared to the oxidative phosphorylation-related genes (Fig. 4B). Transcripts from genes involved in fermentation and the phosphoketolase pathway were relatively less abundant in 12- and 18-hour biofilms, while being relatively more abundant in late-stage biofilms. Specifically, the 24- to 30-hour biofilm timepoints have increased mRNA abundance for genes involved in ethanol, butanediol, and lactate fermentation pathways and the phosphoketolase pathway. Our data also indicate that after 12 hours, there is a decrease in transcripts associated with branched-chain amino acid metabolism pathways. These data suggest that developed biofilms enter a state of reduced oxidative phosphorylation and potentially rely on fermentative metabolism for redox homeostasis during biofilm maintenance.

### Metabolic flux analysis supports fermentative shifts in biofilms

While transcriptomics data revealed increased expression of genes involved in fermentation during biofilm development, gene expression alone does not directly inform changes in metabolic activity. To test whether metabolic pathway activity shifts during biofilm development, we developed a genome-scale metabolic model for strain CEA10 and used experimental data to constrain the model for metabolic flux inference. The CEA10 model was reconstructed and manually curated from a published pan-genome model (14) with several major refinements, including update of biomass composition coefficients, addition of ethanol and butanediol fermentation, and correction of the electron transport chain (ETC) proton gradient, ATP synthesis, and constraints of NADH/NAD+ and NADPH/NADP+ redox reactions (see Materials and Methods for details). The model was constrained by a wealth of experimental data, including specific biomass production rate (Fig. S4), oxygen consumption rate (Fig. S5), and our biofilm transcriptomics data. For transcriptomics data integration, we developed an algorithm to shut down fluxes for reactions associated with lowly expressed genes as much as possible and maximize the correlations between gene expression and fluxes at each timepoint (see Materials and Methods for details). The predicted metabolic fluxes at all timepoints are provided in Table S5.

**Fig 5:**
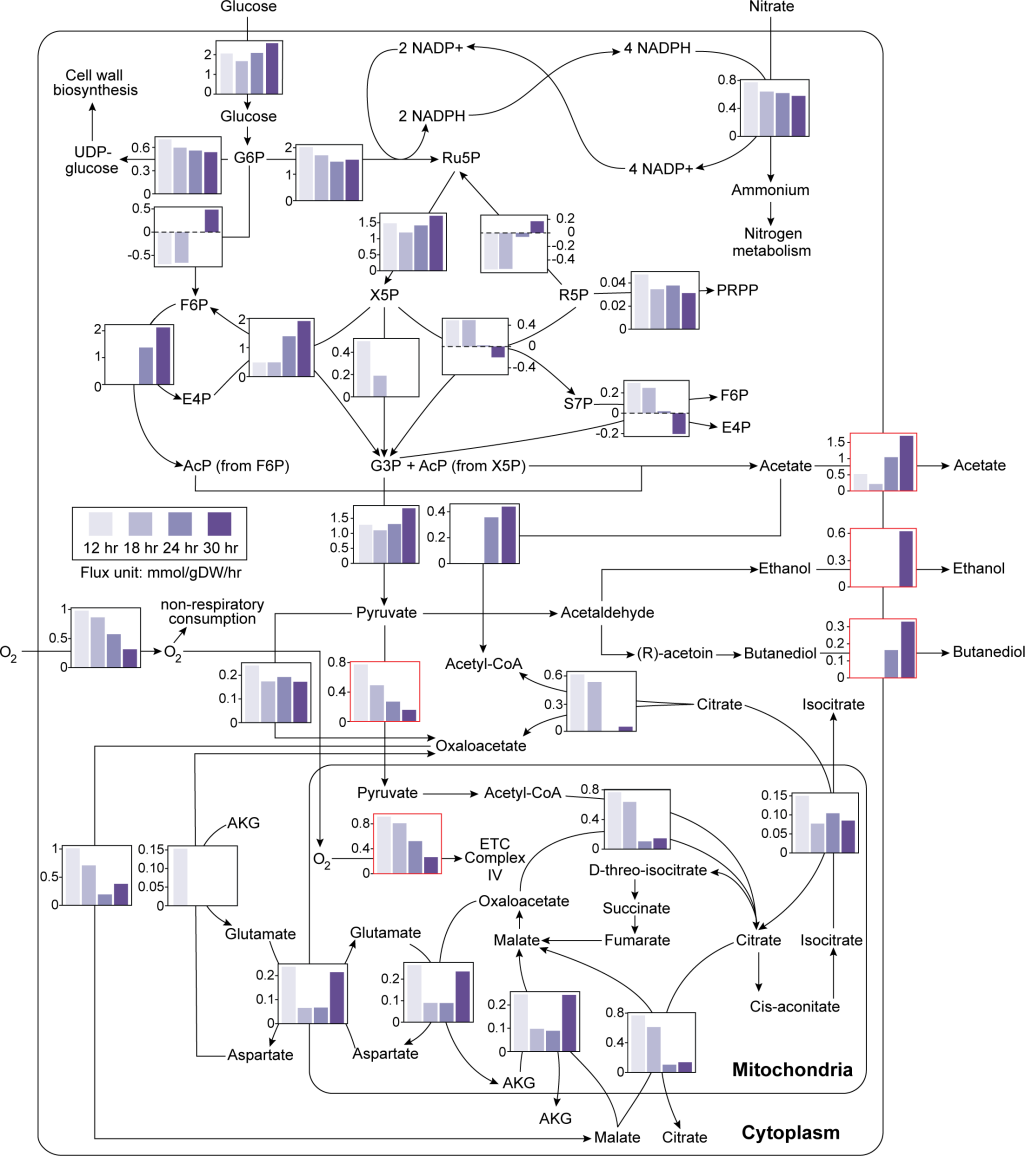
Metabolic shift in *A. fumigatus* CEA10 during biofilm development. Metabolic flux distributions were inferred by constraining a CEA10-specific genome-scale metabolic model with biomass, oxygen, and transcriptomics data (see Materials and Methods for details on the inference algorithm). Selected fluxes are highlighted in red to illustrate the metabolic shift from oxidative phosphorylation to fermentative metabolism with increased production of acetate, ethanol, and butanediol. Abbreviations: glucose-6-phosphate (G6P), fructose-6-phosphate (F6P), glyceraldehyde-3-phosphate (G3P), erythrose-4-phosphate (E4P), ribose-5-phosphate (R5P), ribulose-5-phosphate (Ru5P), xylulose-5-phosphate (X5P), sedoheptulose-7-phosphate (S7P), 5-phospho-α-D-ribosyl 1-pyrophosphate (PRPP), uridine diphosphate glucose (UDP-glucose), acetyl phosphate (AcP), nicotinamide adenine dinucleotide phosphate oxidized/reduced forms (NADP+/NADPH), alpha-ketoglutarate (AKG), and electron transport chain (ETC).

Consistent with the observed transcriptional shifts, the metabolic model predicted increased fluxes in acetate, ethanol, and butanediol fermentation pathways at later timepoints, while the fluxes for oxidative phosphorylation decrease over time (Fig. 5). The model specifically predicted that the pentose phosphate pathway carries high flux to supply NADPH, and the majority of NADPH production was used to synthesize ammonium from nitrate. The pentose phosphate pathway supplied xylulose-5-phosphate and fructose-6-phosphate to the phosphoketolase pathway during early (12- and 18-hour) and late (24- and 30-hour) biofilm growth, respectively. The model also predicted that the phosphoketolase pathway converts acetyl-phosphate to acetate, which was then secreted together with butanediol and ethanol at 24 and 30 hours to maintain NAD+ levels for glycolysis. To assess the robustness of these results independent of the increased phosphoketolase activity, we computationally inactivated the phosphoketolase pathway by deleting the two putative phosphoketolases in *A. fumigatus*, AFUB_038370 and AFUB_048080. The phosphoketolase-null model rewired glycolytic fluxes to the traditional Embden-Meyerhof-Parnas pathway through phosphofructokinase, abolished acetate secretion, and showed comparable high fluxes in the butanediol and ethanol fermentation pathways (Table S5). This *in silico* knockout experiment demonstrates the robustness of the metabolic shift from oxidative phosphorylation to fermentation at late-stage biofilm growth.

### Fermentation is functionally important for biofilm development

Both transcriptomic data and metabolic flux analyses are consistent with a shift from respiration at early timepoints to fermentation at later timepoints. We subsequently tested the hypothesis that fermentation pathways are important for biofilm maturation and/or maintenance through the generation of null mutants of genes involved in two fermentative pathways: ethanol and butanediol metabolism.

**Fig 6:**
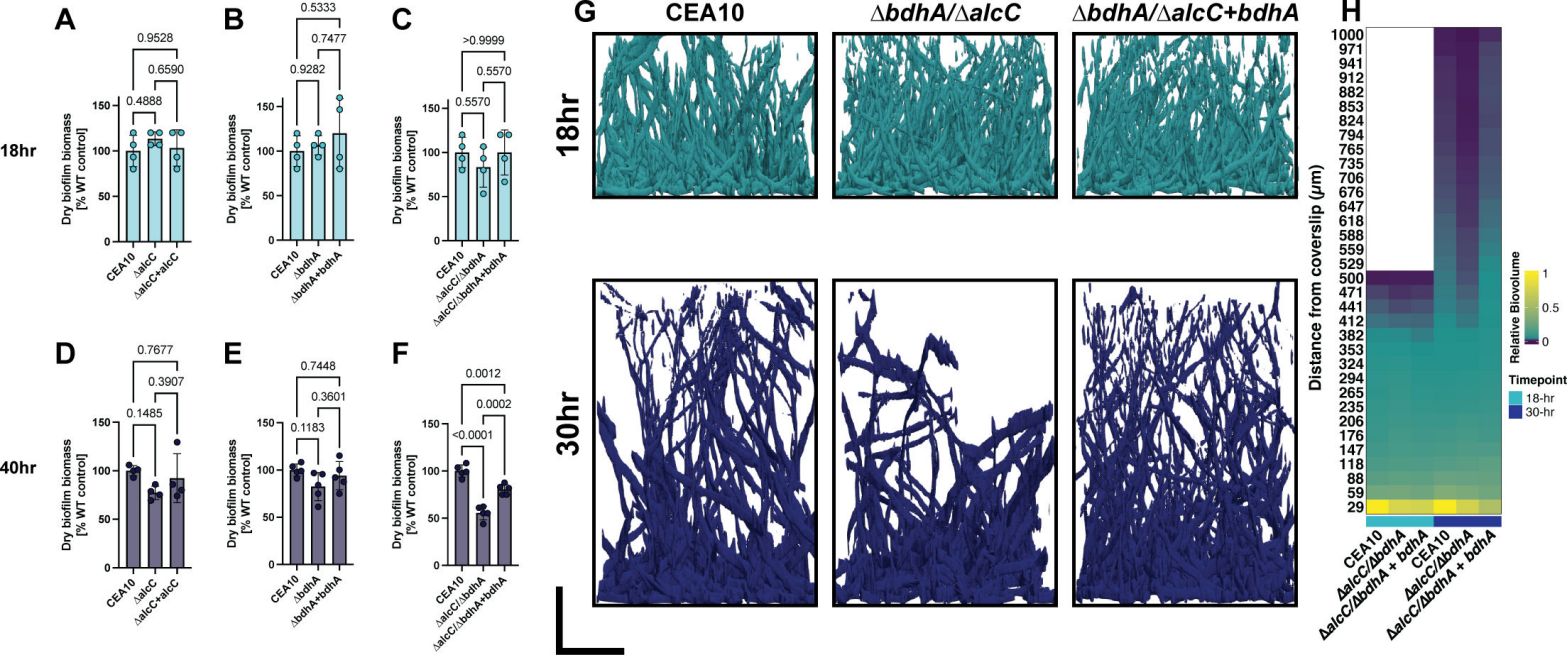
Fermentation metabolism is important for biofilm maturation. (**A–C**) Biofilm biomass of fermentation mutants at 18 hours of growth. (**D–F**) Biofilm biomass of fermentation mutants at 40 hours of growth. Points indicate biological replicates (averages of two technical replicates) with an *n* = 4–5 biological replicates. Statistical analysis is an ordinary one-way ANOVA with a Tukey’s multiple comparison test. (**G**) Rendered representative microscopy images of fermentation mutant biofilms acquired at 18 and 30 hours of growth. The scale bar is 200 µm. (**H**) Heatmap of biovolume quantification of microscopy images. Data represent the average of three biological replicates (each an average of three technical replicates).

Our group previously investigated the alcohol dehydrogenase involved in ethanol production, AlcC, observing that loss of *alcC* resulted in a reduction in fungal burden in a murine model of invasive pulmonary aspergillosis (IPA [15]). The cause for the reduction in fungal burden of the ∆*alcC* strain remains ill-defined, and the role of AlcC in biofilm formation was not explored. One possibility given *alcC* transcript dynamics in the submerged biofilm model is that loss of *alcC* impacts the ability of the fungus to form or maintain a mature biofilm. To test this hypothesis, we quantified biofilm biomass of the ∆*alcC* strain at early (18-hour) and late (40-hour) biofilm timepoints (Fig. 6A and D). The CEA10 ∆*alcC* strain did not have a statistically significant difference in overall biomass at early- or late-stage biofilms.

Given that our transcriptional and metabolic flux analyses indicate that multiple putative fermentation pathways are induced in the biofilm at later stages, it is possible that loss of a single fermentation pathway in our model does not substantially impact biofilm integrity. We hypothesized that removing one pathway would shift the metabolic flux to other fermentative pathways. In support of our hypothesis, we observed that the ∆*alcC* mutant has an increased production of acetoin, an intermediate product of the butanediol fermentation pathway, when shaking batch cultures grown in atmospheric oxygen conditions are shifted to low-oxygen conditions for 48 additional hours (Fig. S6A and D). Despite the transcript profile suggesting an increase in butanediol production in our submerged biofilm model, we were unable to detect acetoin from the submerged biofilm cultures (data not shown). It is possible that the low culture biomass in our model precludes the detection of fermentation products in the culture medium.

We consequently impaired the butanediol fermentation pathway by generating a null mutant for the gene encoding the putative butanediol dehydrogenase AFUB_031610, herein named *bdhA*. We identified this gene and encoded amino acid sequence in the *A. fumigatus* genome as the top hit in a reciprocal blast search for the *Saccharomyces cerevisiae* Bdh1p sequence. An EMBOSS Needle alignment between AFUB_031610 and Bdh1p revealed 36.4% identity and 56.7% similarity (16). Similar to the ∆*alcC* strain, the ∆*bdhA* strain did not have a statistically significant defect in biofilm integrity (Fig. 6B and E). We did not detect acetoin accumulation in the ∆*bdhA* strain, providing support that the ethanol production pathway is the preferred *A. fumigatus* fermentation pathway (Fig. S6B and D). We next impaired both the ethanol and butanediol fermentation pathways by generating a double null mutant strain, ∆*bdhA/∆alcC*. We tested this strain for biofilm formation at 18 and 40 hours. At 18 hours, no significant difference was observed between the double mutant and the WT. However, at 40 hours, the double mutant had a statistically and biologically significant ~50% reduction in biofilm biomass compared to WT control, strongly indicating that these two fermentation pathways are important for wild-type (WT) biofilm integrity in this model (Fig. 6C and F). Restoration of *bdhA* expression in the double mutant restored biomass levels to those of the ∆*alcC* strains as expected. Relative biovolume of the respective biofilms was quantitated with confocal microscopy and supports the conclusion that loss of *alcC* and *bdhA* significantly reduces biofilm integrity at late stages of development (Fig. 6G and H). We quantified acetoin in the ∆*alcC/∆bdhA* strain and found a similar level as the ∆*alcC* strain (Fig. S6C and D). From this finding, we hypothesize that other fermentative pathways derived from pyruvate, such as lactate fermentation, have increased flux when both ethanol and 2,3-butanediol fermentation pathways are impaired. In support of this hypothesis, *in silico* running of our metabolic model with *alc*C and *bdhA* impaired led to an increase in lactate production by 30 hours (Table S5). Taken together, these data support the transcriptional and *in silico* metabolic flux data generated hypothesis that fermentation pathways are critical for *A. fumigatus* biofilm development.

### Transcription factor transcript abundance is dynamic during biofilm development

**Fig 7:**
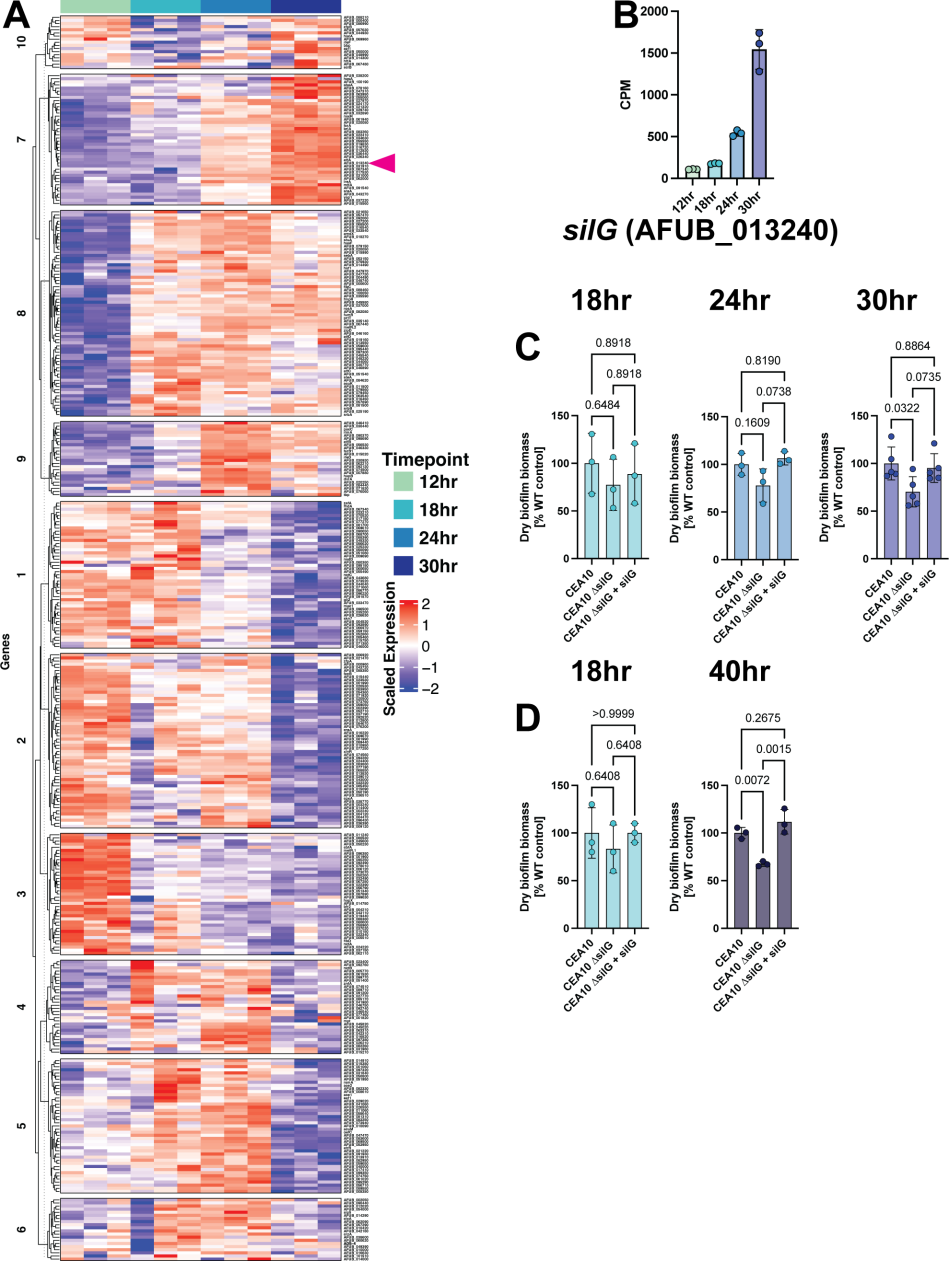
The predicted transcription factor *silG* is involved in biofilm development. (**A**) Heatmap showing transcript abundance of 429 transcription factors with hierarchical clustering and 10 k-means clusters (indicated by number to the left of the heatmap). (**B**) Transcript abundance of the transcription factor *silG* (AFUB_013240) during development (also indicated by pink arrowhead in heatmap). (**C**) The ∆*silG* null mutant is impaired in biofilm development at later stages of development. Biofilm biomass of the ∆AFUB_013240 mutant at 18-, 24-, and 30-hour timepoints. (**D**) The ∆*silG* mutant has a further reduction in biofilm biomass production at 40 hours of growth. Dots represent biological replicates, which are averages of two technical replicates with *n* = 3–4 biological replicates. Statistics shown are a one-way ANOVA with a Tukey’s multiple comparison test.

We were next interested in whether genes predicted to encode transcription factors and kinases were dynamically regulated in the developing submerged biofilm model. We subset our data for the 429-transcription factor and 108 kinase genes found in the COFUN transcription factor null mutant collection and utilized hierarchical clustering of the transcript abundances of these genes over biofilm development (17, 18). From this, we found 10 K-means clusters that sufficiently separate out the transcription factors and 4 clusters that separate out the kinases based on the similarity of their expression profiles (Fig. 7A; Fig. S7). We chose to focus on the transcription factors to explore direct regulators of gene expression in this data set. We observed several clusters of transcription factors that have dynamic transcript abundance regulation during biofilm development. For example, clusters 7, 8, and 9 have low transcript abundance in early timepoints and high transcript abundance in later timepoints, while clusters 1, 2, and 3 have high transcript abundance in early timepoints and low transcript abundance in later timepoints.

### A biofilm development-responsive transcription factor

Through our analysis of transcription factor transcript dynamics, we identified a potential regulator of biofilm development and maintenance, an uncharacterized putative transcription factor, AFUB_013240. AFUB_013240 transcript levels are significantly increased throughout the course of biofilm development, with its highest transcript levels observed in 30-hour biofilms (Fig. 7B). AFUB_013240 was also found among the “step-up” genes identified above (Fig. 3E). Furthermore, AFUB_013240 transcripts were also observed to be significantly increased in response to low-oxygen conditions in two previous studies (19, 20). A reciprocal protein BLAST search for orthologs in *S. cerevisiae* revealed that the closest yeast homolog to AFUB_013240 is the proteasome activator RPN4 (YDL020) (21, 22). However, sequence alignment using the EMBOSS Needle algorithm results in only 16.5% identity and 27.8% similarity between the *A. fumigatus* protein and yeast RPN4p, largely in the C-terminal zinc finger domain. The ortholog to AFUB_013240 in the model mold *Aspergillus nidulans* is the repressor of sexual development *silG* (AN0709) with 56.3% identity and 67% similarity throughout the protein using EMBOSS Needle alignment (23). The molecular function and biological processes associated with AFUB_013240 remain to be clearly defined, but we herein name AFUB_013240 *silG* consistent with the gene name given in *A. nidulans*.

### SilG plays a role in biofilm development

Given the high levels of expression of *silG* in late-stage biofilms, we hypothesized that *silG* plays a role in biofilm development. To investigate this, we generated a *silG* null mutant strain by replacing the AFUB_013240 coding sequence with the hygromycin B resistance gene *hphB* in the strain CEA10. Given the previously observed low-oxygen induction of *silG,* we first investigated whether *silG* was essential for low-oxygen fitness utilizing standard colony biofilm growth assays at 21% and 0.2% O_2_. Loss of *silG* did not impact radial growth or colony morphology under the tested low-oxygen conditions compared to the WT strain, indicating that *silG* does not play a role in low-oxygen colony biofilm growth or morphology (Fig. S8).

We next assessed the impact of *silG* loss in the submerged biofilm model, where we observed a striking time-dependent increase in silG transcript levels over time. Consistent with the transcript profile, the ∆*silG* strain has equivalent biofilm biomass to the WT and reconstituted strains at early timepoints (18 and 24 hours) but has a reduction in biomass in late-stage biofilms (Fig. 7C). This reduction is significant at the 30-hour timepoint, when *silG* expression is the highest. We additionally investigated the biomass of the ∆*silG* mutant at the 40-hour timepoint and observed a continued trend of reduced biofilm biomass (Fig. 7D). Therefore, we conclude that *silG* plays a role in the development and maintenance of *A. fumigatus* biofilms.

## DISCUSSION

Prior to our current study, the transcriptional landscape of the *A. fumigatus* submerged biofilm model was not described. Our understanding of the biology of this organism at a transcriptional level has come from shaking batch cultures and colony biofilm modes of growth (19, 20, 24–26). However, neither of these modes of growth closely recapitulates the structure and environment of *A. fumigatus* growth *in vivo*. For example, colony biofilms on solid medium rapidly induce asexual development, which is generally not observed during mammalian infection; and batch cultures contain homogeneous environments often with pellet-like fungal growth, also not observed *in vivo*. We have previously observed that our *A. fumigatus* submerged culture biofilm model establishes a similar fungal structure as observed in murine models of IPA and human lung histopathology samples (3, 15, 19). Support for this model’s relevance to discovering *in vivo* essential genes includes the observation that the *srbA* null mutant fails to form a biofilm in this model and *in vivo,* and the herein observed impact of fermentation pathways on biofilm formation (3, 15, 27, 28). Moreover, the model was successful in identifying the alanine aminotransferase, *alaA,* as a mediator of echinocandin susceptibility in biofilms and in a murine model of IPA (29).

Utilizing an RNA-Seq approach to define transcript abundance at selected timepoints of *A. fumigatus* biofilm development revealed temporally unique transcriptional profiles. A hallmark of these temporally distinct transcript profiles is dynamic changes in transcripts associated with metabolism. By looking directly at transcripts associated with primary metabolism, we find a shift in the transcript levels of genes associated with respiration (oxidative phosphorylation) and fermentation as the biofilm matures (Fig. 4). These transcriptomic observations were supported by constraint-based metabolic modeling, which predicted reduced flux through oxidative phosphorylation and increased flux through ethanol, butanediol, and acetate fermentation pathways during biofilm development (Fig. 5). Although this is the first time such a shift has been described in an *A. fumigatus* submerged biofilm model, the observation of metabolic shifts as the result of a biofilm mode of growth has been previously described in bacterial and other fungal biofilm systems (30–33). Similar to our model system here, in these systems, there are metabolic shifts that occur during biofilm development and maturation. In our model fungal biofilm system, the shift is away from respiration to favor fermentation. The mechanism(s) driving this metabolic shift are unclear; our modeling suggests that the shift is driven in part by oxygen consumption; however, changing micronutrient and macronutrient availability within the biofilm structure as development progresses likely also plays a significant role. Importantly, considering the microenvironmental changes occurring within biofilms in this model raises a potential limitation; the primary carbon and nitrogen sources utilized by the fungus *in vivo* remain ill-defined. Thus, our data must be interpreted recognizing the use of glucose and nitrate as the sole carbon and nitrogen sources, respectively, for biofilm development in the studied model.

In further support of the model’s *in vivo* relevance, a previous study identified the alcohol dehydrogenase, AlcC, as a mediator of fungal burden in a murine model of IPA (15). The mechanism driving the reduction of fungal burden *in vivo* in the absence of *alcC* has remained ill-defined. Here in our analysis, we observed increases in transcripts associated with fermentation, including *alcC,* as biofilms developed and matured. Thus, one possibility for the reduction of fungal burden associated with loss of *alcC* is an inability to fully develop a mature biofilm in the murine lung environment. While loss of the alcohol dehydrogenase *alcC* did not significantly reduce biofilm biomass in the conditions we examined here, additional loss of the *bdhA* gene involved in butanediol fermentation significantly reduced fungal biofilm biomass. Previously, 2,3-butanedione was detected in the headspace of *A. fumigatus* batch cultures in low-oxygen conditions (34). It is possible that the multiple fermentation pathways induced in the *in vitro* submerged model are due to the large amount of glucose utilized as the primary carbon source in these experiments. Moreover, *A. fumigatus* may produce other pyruvate-derived fermentation products, such as lactate and acetate, as indicated by our model. In concordance with this, a significant flux of carbon through the phosphoketolase pathway, which canonically produces lactate and either acetate or ethanol, is predicted in our metabolic model. It will be important to further dissect *in vivo* relevant carbon and nitrogen sources and how they influence specific fungal fermentation pathways in established infections.

One approach to address the complex regulation of metabolism and the associated metabolic products is to define the regulators of these pathways. We examined the transcript levels of 429 predicted transcription factors and observed significant abundance changes during biofilm development. Interestingly, we observed transcription factor encoding genes among our lowland transcripts at the 18-hour timepoint, indicating the transcripts for many regulatory genes are reduced at this early timepoint during development. An abundance of transcription factor transcripts is strongly regulated between the 12- and 18-hour and 24- and 30-hour timepoints (Fig. 7A). These data further highlight that there is an important change occurring between 12 and 18 hours and then again between 24 and 30 hours. These dramatic transcriptional changes could be the driver of principal component 2 (PC2) from the exploratory analysis, where we found that samples separate on PC2 by early/late biofilms and middle-stage biofilms (Fig. 2B). What is driving these transcriptional changes is unclear. However, previous work from our group has shown oxygen gradients play a key role during biofilm development and are potentially involved in driving the PC2 variability (3). A functional analysis using the genes driving PC2 was unfruitful in this determination of the mechanism responsible. Therefore, the transcriptional shifts are likely driven in part by oxygen gradient dynamics at a given timepoint. Moreover, as indicated by the genetic analysis of specific fermentation pathways, alternative carbon sources produced from glucose metabolism are likely also driving transcriptional changes. Taken together, these data also highlight a general limitation in our approach using bulk-RNA-Seq of the biofilm model, as it is likely that substantial spatial heterogeneity exists within the biofilm. However, these data allow hypotheses to be generated and tested using developing single-cell and reporter gene approaches in the biofilm model.

We further tested the predictive power of the data set by examining a specific, unstudied, predicted transcription factor that is regulated in part by oxygen levels, AFUB_013240, herein called *silG*. The transcript abundance for *silG* is significantly increased in hypoxic conditions in a SrbA-independent manner (19, 20, 27). Interestingly, *silG* transcript abundance was also found to be significantly increased after 16 hours when co-cultured with host epithelial cells (35). In the submerged biofilm model, *silG* transcript levels rise over the course of biofilm development, perhaps consistent with the decreasing levels of oxygen previously observed in mature biofilms. However, we cannot rule out that *silG* transcript levels respond to other changing culture conditions that are concomitant with reductions in oxygen levels. Interestingly, loss of *silG* did not impact colony growth or morphology on agar under low-oxygen conditions. However, loss of *silG* in the submerged biofilm model resulted in a reduction of biofilm biomass exclusively at later timepoints. These results may suggest *silG* responds to a metabolic consequence of the fungal hypoxia response and is not a regulator of the hypoxia response *per se*. SilG has not been characterized fully in filamentous fungi to date, though it may be related to the proteasome regulator in yeast, RPN4. It is intriguing that *silG* has been associated with sexual reproduction in *A. nidulans,* which is promoted by oxygen-limiting conditions. Future studies on the function of *silG* in *A. fumigatus* should include analysis of the genes it regulates and its impact on pathogenicity and disease progression in murine models of aspergillosis.

## MATERIALS AND METHODS

### Strains and growth conditions

Mutant strains were made in the *A. fumigatus* CEA10 background, also called FGSC A1163; therefore, CEA10 was used as the WT strain as appropriate for each experiment. Strains were stored as conidia in 25% glycerol at −80°C and grown on 1% glucose minimal medium (GMM) at 37°C (36). Conidia were collected using 0.01% Tween 80.

### Microscopy

Images of biofilms were acquired using a Nikon spinning disk microscope fitted with a Yokogawa CSU-W1 spinning head and a 20× dry objective. Biofilms were grown for the indicated times and stained with 25 µg/mL of calcofluor white 20 minutes prior to imaging. Three-dimensional image rendering was performed using BiofilmQ analysis software and Paraview visualization software (Kitware) (19, 37).

### RNA extraction from biofilm tissue

Submerged liquid cultures were seeded in GMM with 10^5^ spores per mL in 100 mm petri dishes. Biomass was collected via filtration through Miracloth and snap frozen in liquid nitrogen. Frozen biomass was bead-beaten in 200 μL of TRI Reagent (Invitrogen) with 2.3 mm silica beads. Homogenate was brought to a final volume of 1 mL with TRI Reagent, and RNA was extracted by following the manufacturer’s protocol.

### RNA sequencing

RNA for RNA-seq was quantified by Qubit (Thermo Fisher Scientific), and integrity was measured on a fragment analyzer (Agilent). Samples with RIN ≥7 underwent library preparation with the mRNA HyperPrep kit (Kapa Bioscience) using 200 ng RNA as input following the manufacturer’s instructions. Libraries were pooled for sequencing on a NextSeq500 instrument (Illumina), targeting 10M, single-end 75 bp reads/sample.

### RNA-sequencing analysis

Raw read quality was initially assessed using fastQC (Babraham Bioinformatics group) and multiQC software (38). Reads were trimmed using Cutadapt software using a quality score cutoff of 20 and again assessed for quality using FastQC and MultiQC (39). Alignments were performed using Star alignment software with the *A. fumigatus* CEA10 reference genome version FungiDB-52 and a general feature format file from the same version (40). Counts per gene were compiled using HTSeq software (40, 41).

For the exploratory analysis, the package DESeq2 was used to generate vst normalized values for use in clustering and principal component analysis (42). The package edgeR was used to normalize read data for use in differential expression analysis using the TMM method (43). Low-abundant transcripts were filtered using the filterByExpr function, and normalization was performed using the CalcNormFactors with trimmed mean of the M-values. Limma-voom methodology was used to perform a differential expression analysis using linear model fit with Empirical Bayes moderated t-statistics and multiple comparisons between all pairwise timepoint combinations (44). Scaled CPM values are used for heatmap representation using the R scale function, which works by subtracting values from the mean CPM for each gene and dividing by the standard deviation for the same gene. Heatmaps were generated using the ComplexHeatmap package (45).

### Overview of metabolic modeling and flux analysis

The genome-scale metabolic model for *A. fumigatus* CEA10 was constructed and analyzed using COBRApy (46) and the IBM CPLEX solver (version 22.1.1). By constraining the model with specific biomass production rates, oxygen consumption rates, and transcriptomics data, we predicted the dynamic changes in metabolic fluxes across *A. fumigatus* biofilm development over a 30-hour window. Details of the model reconstruction and metabolic flux analysis are described below and also available in the supplemental computer codes (see Data availability statement).

### Construction and refinement of a CEA10 genome-scale metabolic model

The draft CEA10 model was constructed using a previously published protocol (14). Briefly, the model was pruned from the *A. fumigatus* pan-genome model (14) by mapping the CEA10/A1163 genome to the pan-genome annotation and retaining CEA10-specific genes (at least 95% sequence identity) and associated reactions. The A1163 genome is available through The Central Aspergillus Data Repository (47). Gaps in metabolic pathways were filled using the minimal cut set approach described in the protocol. We confirmed that the gap-filled draft model can grow in glucose minimal medium.

The draft CEA10 model was manually curated and refined through the following steps. The biomass reaction was corrected using the measured molar biomass composition (14). This correction substantially increased the model’s maximum yield, bringing it in line with observed values (~0.5 g dry biomass per g glucose) in glucose minimal medium under normoxic planktonic conditions. The ETC reactions were edited to reflect a proton gradient generating 10 H+ across the full ETC and a maximum ATP yield of 2.5 per NADH and 1.5 per FADH_2_ (formerly 1 ATP each) by adding proton translocation to Complex I and Complex III. Additionally, reactions that produce oxygen and allow biomass production in anaerobic conditions (e.g., the phenol:oxygen oxidoreductase reaction KEGG: EC 1.14.13.7) were systematically identified and made irreversible in the oxygen-consuming direction.

The draft model contains unrealistic reaction loops that artificially convert NADH into NADPH by allowing the same substrate to be oxidized and reduced with different cofactors. For such coupled reactions, we manually curated the cofactor specificity. Enzyme variants that could unrealistically use NADP+ were either removed or modified to use the biologically correct cofactor (e.g., acetaldehyde dehydrogenase was constrained to use NAD+). These corrections produced the expected behavior, with NADPH generated through the pentose phosphate pathway for biosynthesis and NAD+ regenerated through fermentation to maintain glycolysis. Ethanol production was achieved in the model by allowing the NADH:acetaldehyde redox reaction catalyzed by alcohol dehydrogenase to operate reversibly. We further incorporated the butanediol fermentation pathway, which was absent in the draft model, by adding the final step of acetoin reduction catalyzed by the putative butanediol dehydrogenase *bdhA* and other necessary transport reactions.

Acetate kinase reactions were revised to use ATP/ADP exclusively, consistent with their activity in both bacteria and *Cryptococcus neoformans* (48, 49). Passive diffusion of acetyl-CoA from mitochondria to the cytosol was blocked. Instead, acetyl-CoA can be generated from citrate, which is transported between compartments through the citrate shuttle system, such as the citrate-isocitrate transporter and the malate-citrate transporter. We also added a mitochondrial malate-alpha-ketoglutarate antiporter reaction to enable the malate-aspartate shuttle. Finally, the gene-protein-reaction (GPR) rules for NADPH:nitrate oxidoreductase and mitochondrial citrate synthase were updated to include the primary genes for these enzymes, that is, *niaD* (AFUB_012300) and *cit1* (AFUB_052750), respectively, based on 100% sequence identity with AF293 genome sequences in FungiDB. The code implementing these refinements is available on GitHub (see Data availability statement).

### Quantitative analysis of biomass growth and oxygen consumption

Biofilm biomass production rates were determined by simultaneously fitting the OD curve and biomass data using a modified exponential function with growth delay (Fig. S4)


$$y=\left\{ \begin{array}{ll} b_{0} & t<t_{0}\\ b_{0}e^{r{\left(t-t_{0}\right)}^{n}} & t\geq t_{0} \end{array}\right.$$

where $b_{0}$ represents the basal OD or biomass, $t_{0}$ represents the growth delay, r represents the specific growth rate (per-biomass growth rate), and n is a scaling factor that captures the deviation from simple exponential growth (i.e., n=1). The best-fit parameter values are $b_{0}=0.024$ for OD and 0.424 for biomass, $t_{0}=10.336\;hours$, $r=0.170{\left(hr\right)}^{-n}$, and n=0.893. The specific growth rates at 12, 18, 24, and 30 hours were determined from (1/y)(dy/dt) at the corresponding timepoints. The best-fit scaling factor n is less than 1, indicating reduced specific growth rate over time.

The oxygen consumption rates at 12, 18, 24, and 30 hours were estimated by fitting a mathematical model to the dissolved oxygen concentrations we previously measured at the lowest depth (3). The model has two terms that describe oxygen consumption by *A. fumigatus* cells and oxygen diffusion after $t\geq t_{0}$ (oxygen was assumed not to be consumed before $t_{0}$):


$$\frac{d\left[O_{2}\right]}{dt}=\frac{D*A}{x*V}*\left(O_{2,sat}-\left[O_{2}\right]\right)-\frac{V_{max}*\left[O_{2}\right]}{\left[O_{2}\right]+K_{m}}*\left[Biomass\right]$$

In this equation, $\left[O_{2}\right]$ represents the oxygen concentration, D represents the coefficient of diffusion, A represents the surface area of the well ($9.6\;cm^{2}$), x represents the distance of diffusion (1.5 mm), V represents the volume occupied by the biomass (1 mL), within which oxygen is assumed to be uniformly distributed, $O_{2,sat}$ represents the saturated oxygen concentration at 37°C (200 μM), $V_{max}$ represents the maximum oxygen consumption rate by *A. fumigatus* cells, and $K_{m}$ represents the oxygen concentration at half-maximum consumption rate. Notably, these values reflect bulk biofilm activity and do not account for the spatial heterogeneity in growth and nutrient availability present in the biofilm. The biomass concentrations (Biomass) were modeled using the previously determined best-fit growth equation.

The diffusion constant D was set either to the literature static diffusion coefficient ($2.624*10^{-5}\;cm^{2}/s$ [50]) or to an effective value that accounts for both diffusion and convective effects caused by hyphal growth and metabolic activity ($8.667*10^{-5}\;cm^{2}/s$ [3]). Both diffusion constants fit the oxygen concentration data equally well (Fig. S5A); however, they result in substantially different estimates of oxygen consumption rates (Fig. S5B). We selected the convecting diffusion coefficient because it captures the delayed onset of hypoxia and provides a conservative estimate of fermentation flux. Using this diffusion constant, we determined the values of free parameters as 1.156 1/hour for $V_{max}$ and 0.024 mM for $K_{m}$. A detailed description of model assumptions and the code used for parameter estimation can be found on GitHub (see Data availability statement).

### Transcriptomics-guided metabolic flux inference

At each timepoint, the CEA10 metabolic model was constrained using the specific growth rates and oxygen consumption rates estimated above. The glucose and nitrate uptake rates were set to the minimum values required to satisfy these constraints. With biomass production, glucose, nitrate, and oxygen uptake all constrained, we applied a modified iMAT algorithm (51) to identify the maximal number of reactions that were associated with the lowest 25% gene expression (transcripts per million [TPM] thresholds 2.4–3.5, depending on the timepoint) and carry zero flux. For reactions catalyzed by multiple genes, we determined the specific gene that dictates the reaction activity by analyzing their GPR rules (i.e., the highest expressed gene among functionally redundant “OR” genes, and the lowest expressed gene among “AND” genes forming a multi-subunit or multi-enzyme complex). The glucose and nitrate uptake rates were then adjusted and set to 101% of their respective minimal values required by the iMAT-constrained models. The optimal metabolic flux distribution for these models was obtained by maximizing the gene expression-flux correlation. Specifically, each reaction was assigned a score of $log_{2}\left(TPM/100\right)$, using the TPM of the gene identified as dictating the reaction’s activity. This score penalizes flux through reactions associated with genes expressed below 100 TPM and rewards flux through reactions associated with genes expressed above 100 TPM. Using these scores, the optimization objective is formulated as follows:


$$maximize\sum_{reaction\;i}Score_{i}*|flux_{i}|$$

After the optimization, parsimonious flux balance analysis (pFBA) (52) was used to identify flux distributions that minimize the total sum of reaction fluxes, under all constraints described above, including 95% of the maximal gene expression-flux correlation. These pFBA solutions were taken as the final predicted fluxes for each timepoint.

### Strain construction

The ∆*silG* null mutant was generated by replacing the *silG* open reading frame (AFUB_013240) with the dominant selection marker *hphB* in the CEA10 background, utilizing a CRISPR/Cas9-mediated transformation into protoplasts as previously described (29, 53). The repair construct with the *hphB* resistance marker was amplified using RAC7880 and 7881. This amplified repair was transformed into CEA10 protoplasts using CRISPR/Cas9-mediated targeting to the *silG* locus. Mutants were selected for growth on osmotically stabilized media containing hygromycin. Loss of *silG* was confirmed by Southern blot analysis using DIG-labeled probe generated by primers RAC8065 and RAC8066 against 30 μg of WT and ∆*silG* gDNA digested using EcoRV-HF (NEB) restriction enzyme in CutSmart Buffer (NEB) for 16 hours at 37°C. Digested DNA was run on 1% agarose gel for 2 hours at 80V, transferred to PVDF membrane using a vacuum blotter, and then probed overnight with DIG-labeled DNA probe (Roche Diagnostics, Mannheim, Germany). Antibody detection was done according to the manufacturer’s protocol (Roche). Southern blot was visualized using a chemiluminescent gel imaging system, DIG-labeled Molecular Weight Marker VII was used for DNA ladder (Roche).

Reconstitution of the AFUB_013240 gene was performed by PCR amplification of the AFUB_013240 locus from WT gDNA from ∼1,000  bp upstream of the start codon to ∼1,000 bp downstream of the stop codon using primers RAC8178 and RAC8179 with homology to a backbone plasmid containing the pyrithiamine resistance marker *ptrA*. The backbone plasmid was amplified using primers RAC8180 and RAC8181. The resulting PCR products were assembled using the NEBuilder Hi-Fi DNA assembly kit (NEB). A repair construct was amplified from this resulting plasmid using primers that contained homology to the *aft4* safe-haven locus using primers RAC8184 and RAC8185 (54). The repair construct was transformed into ∆*silG* protoplasts using CRISPR/Cas9-mediated targeting to the *aft4* safe-haven site. Mutants were selected for growth on osmotically stabilized media containing pyrithiamine. Reconstitution of gene expression was validated by RT-qPCR on RNA extracted from 24 hours WT, ∆*silG,* and *silG*^recon^ biofilms using the primers RAC8464 and RAC8465.

The ∆*bdhA* null mutant was generated by replacing the *bdhA* open reading frame (AFUB_031610) with the *pyrG* selection marker in the uridine/uracil auxotrophic CEA17 background. RAC4242 and RAC4239 were used to amplify a ~1.8 kb region 5′ to the *bdhA* open reading frame, and RAC4240 and RAC4243 were used to amplify a ~850 bp region 3′ to the open reading frame. The *pyrG* gene, along with its promoter and terminator, was amplified using RAC2055 and RAC2056. The two flanks and the *pyrG* marker were combined via overlap PCR, and the resulting product was used to transform CEA17 protoplasts as previously described (29). Mutants were selected for growth on osmotically stabilized minimal media lacking uridine/uracil supplementation (GMM plus 1.2M sorbitol). Similarly, to generate the ∆*bdhA*∆*alcC* double mutant, the *bdhA* open reading frame was replaced with a pyrithiamine resistance selection marker in a previously published ∆*alcC* strain (15). RAC2055 and RAC2056 were used to amplify the pyrithiamine selection marker, and the resulting product was combined with the two previously mentioned ~1.8 kb and ~850 bp flanks via overlap PCR. Mutants were selected on osmotically stabilized minimal media containing pyrithiamine. Both *bdhA* null strains were single-spored and checked for correct locus integration via PCR and Southern blotting.

The *bdhA* reconstitution construct was generated by amplifying the *bdhA* ORF ±1,000 bp upstream and downstream from *bdhA* using RAC8921 and RAC8922 and the plasmid backbone containing the *hphA* resistance cassette with RAC8923 and RAC8924. The bdhA cassette was integrated into the plasmid backbone by HiFi DNA assembly using NEBuilder (NEB), resulting in the pbdhA-hphA plasmid. The reconstitution repair construct was PCR amplified from pbdhA-hphA using RAC8964 and RAC8965, which contain homology to the *aft4* safe-haven locus, and transformed into ∆*bdhA* protoplasts as described above. Expression levels were analyzed using RT-qPCR on RNA extracted from 24-hour biofilms using the primers RAC8955 and RAC8957.

All strains were single-spored and checked for correct integration via PCR and Southern blotting. Primer sequences used are available in Table S6. All strains generated in this study are available upon request from the corresponding author.

### RT-qPCR

A sample of 5 µg of RNA was DNase treated with the TURBO DNA-free kit (Invitrogen) according to the manufacturer’s protocol. A sample of 500 ng of DNase-treated RNA was run on an agarose gel to ensure RNA integrity, and 500 ng of DNase-treated RNA was used for cDNA synthesis as previously described (55). The RT-qPCR data were collected on a CFX Connect real-time PCR detection system (Bio-Rad) with CFX Maestro Software (Bio-Rad). Gene expression was normalized to *actA* and *tefA* expression for all experiments. Primers used are available in the primer table (Table S1).

### Growth assays

Agar plates were inoculated with 10^3^ conidia and incubated for 72 h at 37°C, 5% CO_2_ in either ambient oxygen or in a chamber that maintained oxygen at a concentration of 0.2% using an InvivO_2_ 400 Workstation (Ruskinn Baker).

Biofilm biomass cultures were inoculated with 2 mL of 10^5^ conidia/mL in GMM and grown in six-well tissue culture plates for the indicated time (12, 18, 24, or 30 hours) at 37°C, 5% CO_2_. Supernatants were removed, and biofilms were harvested via transfer to conical tubes and centrifugation. Biomass was washed 2× with double-distilled water, frozen at −80°C, lyophilized, and the dry weight was measured. For the 40-hour timepoints, 4 mL of 5 × 10^4^ conidia/mL was used to prevent the formation of growth at the air-liquid interface. For 40-hour experiments, parallel 18-hour biofilms were also grown in 4 mL of 5 × 10^4^ conidia/mL to keep comparisons consistent between two timepoints.

### Acetoin quantification with modified Voges-Proskauer assay

Strains were grown in GMM with 5 × 10^7^ conidia in 100 mL, 200 rpm, 37°C, and 5% CO2, for 24 hours. Fungal biomass was collected and swapped into 100 mL of fresh GMM and cultured for 48 hours in hypoxia, 1% oxygen (200 rpm, 37°C, 5% CO2). Mycelia were harvested, rinsed with distilled water, frozen, and lyophilized. Culture supernatants were collected. Acetoin standards (0–1.0 mM) as well as experimental samples were incubated with 0.5% creatine, 5% alpha-naphthol, and 40% potassium hydroxide for 15 minutes. Absorbance was measured at 560 nm, and the mM concentration of acetoin was calculated based on the standard curve (56, 57).

### Statistical analysis

All statistical analyses were performed in GraphPad Prism 10. Error bars indicate standard deviation around the mean.

## Data Availability

The data discussed in this publication have been deposited in NCBI’s Gene Expression Omnibus (58) and are accessible through GEO Series accession number GSE298422. Customized Python codes (version 3.10.18) for metabolic modeling and flux analysis are available on GitHub (https://github.com/nathde22/A_fug_biofilm_metabolic_model).

## SUPPLEMENTAL MATERIAL

Fig. S1 msphere.00752-25-s0001.pdf Genes ordered by variance and PCA.

Fig. S2 msphere.00752-25-s0002.pdf Heatmap of secondary metabolism and related pathways.

Fig. S3 msphere.00752-25-s0003.pdf Heatmap of secondary metabolite biosynthetic gene clusters transcript abundance.

Fig. S4 msphere.00752-25-s0004.pdf Model fit of biofilm growth to experimental data.

Fig. S5 msphere.00752-25-s0005.pdf Model fit to oxygen level at the 2500 μm depth of *A. fumigatus* biofilm.

Fig. S6 msphere.00752-25-s0006.pdf Acetoin accumulation in culture supernatants of low oxygen cultures.

Fig. S7 msphere.00752-25-s0007.pdf Heatmap of kinase transcript abundance with hierarchical clustering.

Fig. S8 msphere.00752-25-s0008.pdf AFUB_013240 is dispensable for colony biofilm growth in hypoxia.

Supplemental Tables msphere.00752-25-s0009.xlsx Tables S1 to S6.

ASM does not own the copyrights to Supplemental Material that may be linked to, or accessed through, an article. The authors have granted ASM a non-exclusive, world-wide license to publish the Supplemental Material files. Please contact the corresponding author directly for reuse.
