# Supplementary material for: Transcriptional and metabolic modeling analyses of developing Aspergillus fumigatus biofilms reveal metabolic shifts required for biofilm maturation
Source: mSphere. 2025 Nov 28;10(12):e00752-25. doi: 10.1128/msphere.00752-25 (PMC12724364; doi:10.1128/msphere.00752-25)
Supplement: Fig. S1 — Genes ordered by variance and PCA. [file msphere.00752-25-s0001.pdf]

Figure S1

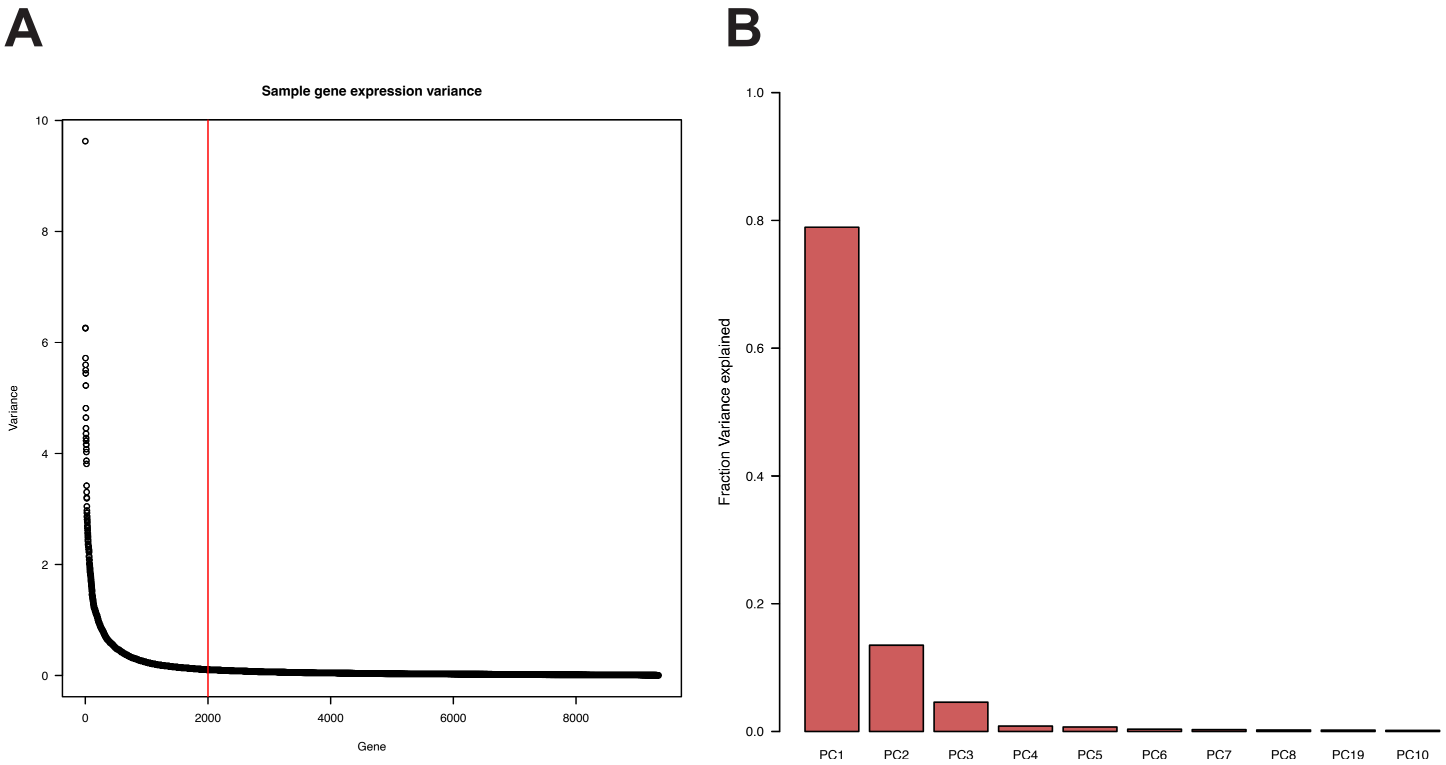

Figure S1: A) Genes were ordered by variance to identify a threshold of 2000 genes to use for PCA and variable gene expression heatmap. **B**) Principal component analysis reveals the first 2 principal components capture most of the variation.
