# Supplementary material for: Transcriptional and metabolic modeling analyses of developing Aspergillus fumigatus biofilms reveal metabolic shifts required for biofilm maturation
Source: mSphere. 2025 Nov 28;10(12):e00752-25. doi: 10.1128/msphere.00752-25 (PMC12724364; doi:10.1128/msphere.00752-25)
Supplement: Fig. S2 — Heatmap of secondary metabolism and related pathways. [file msphere.00752-25-s0002.pdf]

Figure S2

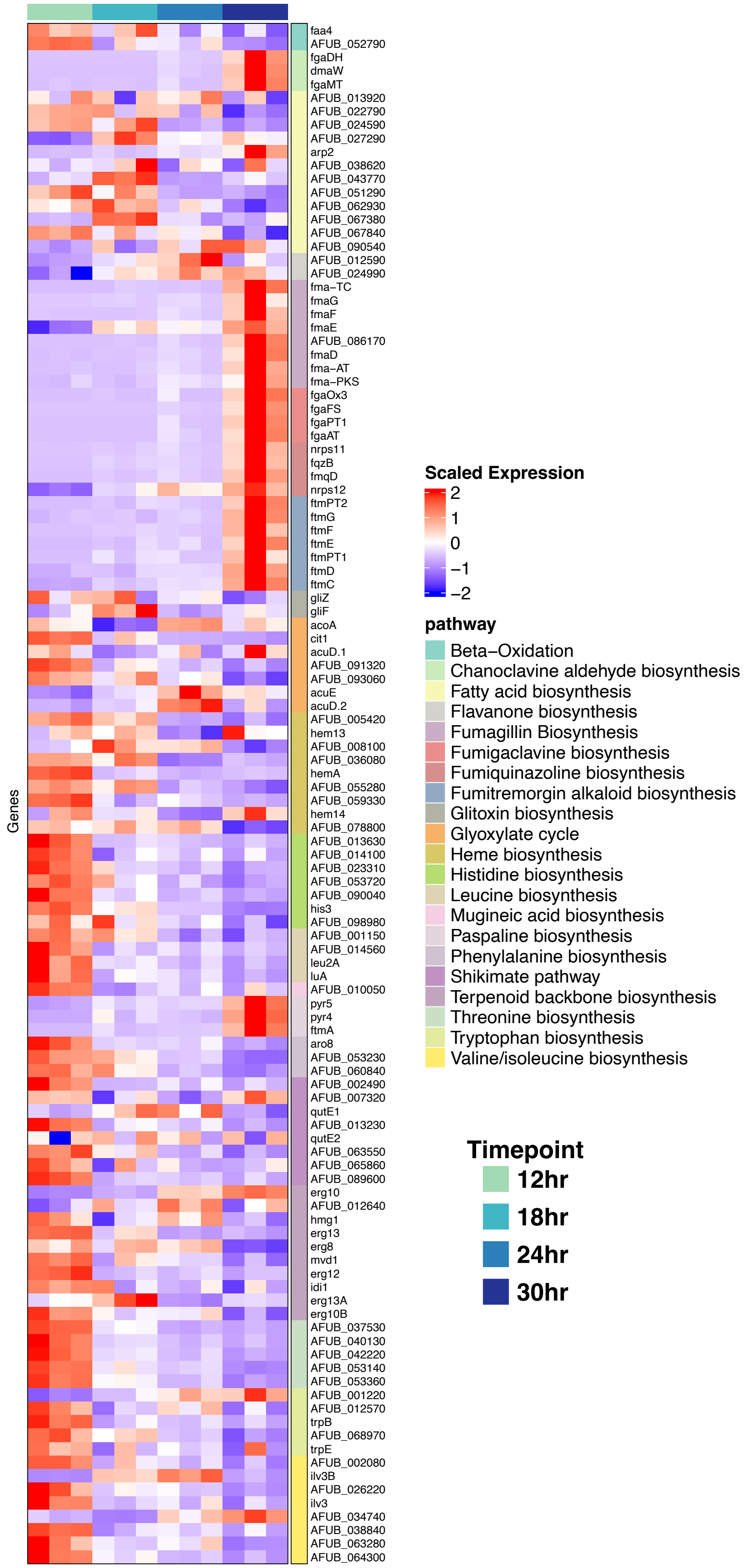

Figure S2: A heatmap of secondary metabolism and related pathways. Pathways were identified from the KEGG database. Pathways are indicated by color in the key and scaled CPM values are shown.
