# Supplementary material for: Transcriptional and metabolic modeling analyses of developing Aspergillus fumigatus biofilms reveal metabolic shifts required for biofilm maturation
Source: mSphere. 2025 Nov 28;10(12):e00752-25. doi: 10.1128/msphere.00752-25 (PMC12724364; doi:10.1128/msphere.00752-25)
Supplement: Fig. S3 — Heatmap of secondary metabolite biosynthetic gene clusters transcript abundance. [file msphere.00752-25-s0003.pdf]

Figure S3

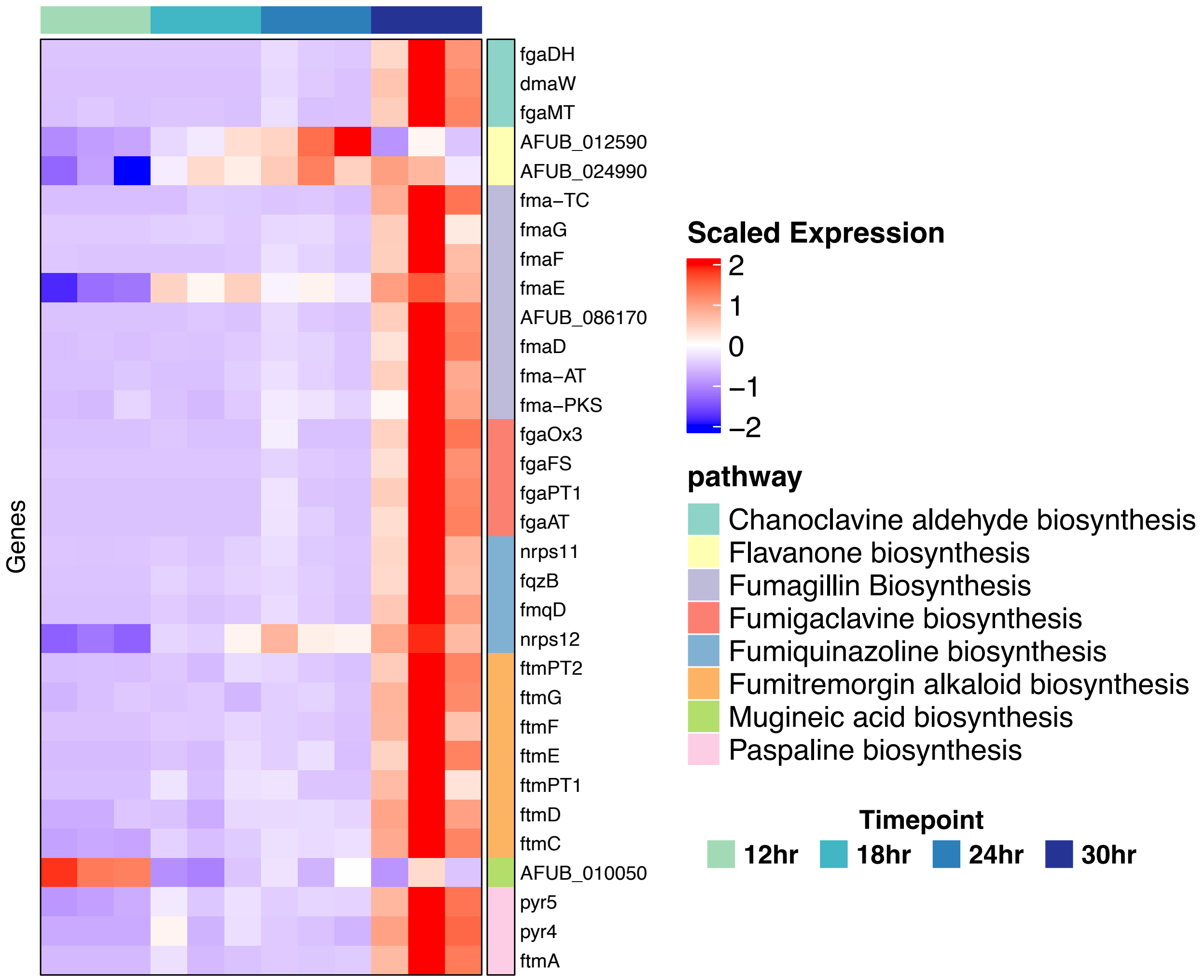

Figure S3: A heatmap of secondary metabolite biosynthetic gene clusters transcript abundance. Several of these gene clusters are up-regulated in late stages of biofilm development. Pathways are indicated by color in the key and scaled CPM values are shown.
