# Supplementary material for: Transcriptional and metabolic modeling analyses of developing Aspergillus fumigatus biofilms reveal metabolic shifts required for biofilm maturation
Source: mSphere. 2025 Nov 28;10(12):e00752-25. doi: 10.1128/msphere.00752-25 (PMC12724364; doi:10.1128/msphere.00752-25)
Supplement: Fig. S4 — Model fit of biofilm growth to experimental data. [file msphere.00752-25-s0004.pdf]

Figure S4

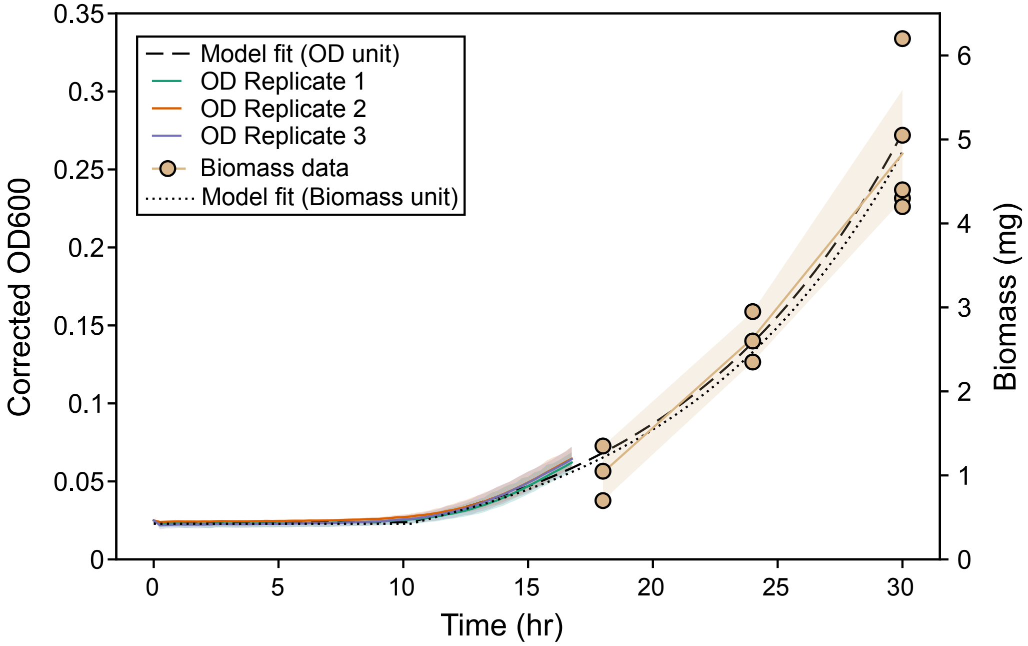

Figure S4: Model fit of biofilm growth to experimental data. A mathematical model was developed to simultaneously fit biofilm optical density (OD) and biomass measurements (see **Methods** for details on model construction and parameter estimation). Experimental data are shown together with model predictions, with the left axis representing OD values and the right axis representing biomass values. The model successfully captures the dynamics of both OD and biomass during biofilm development, which demonstrates its ability to reconcile the two complementary readouts of biofilm growth.
