# Supplementary material for: Transcriptional and metabolic modeling analyses of developing Aspergillus fumigatus biofilms reveal metabolic shifts required for biofilm maturation
Source: mSphere. 2025 Nov 28;10(12):e00752-25. doi: 10.1128/msphere.00752-25 (PMC12724364; doi:10.1128/msphere.00752-25)
Supplement: Fig. S5 — Model fit to oxygen level at the 2500 μm depth of A. fumigatus biofilm. [file msphere.00752-25-s0005.pdf]

Figure S5

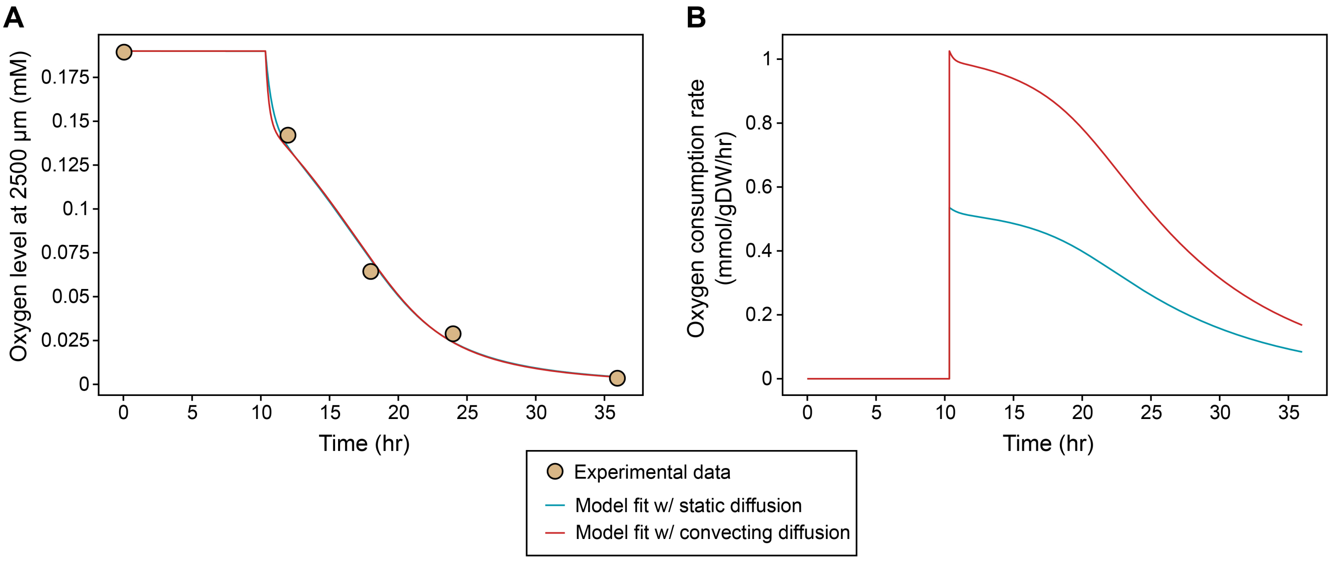

Figure S5: Model fit to oxygen level at the 2500 µm depth of *A. fumigatus* biofilm. Our mathematical model incorporates biofilm oxygen consumption and oxygen transport through both diffusion and convection (see Methods for details on model construction and parameter estimation). We simulated oxygen concentration dynamics using two effective diffusion rate constants representing diffusion alone (no convection) and diffusion combined with convection. **A**) Measured oxygen concentrations overlaid with the best-fit model predictions. **B**) Oxygen consumption rate inferred from the best-fit models. While both diffusion constants fit the measured oxygen data equally well, the inferred oxygen consumption rates differ, with convecting diffusion resulting in higher consumption rates.
