# Supplementary material for: Transcriptional and metabolic modeling analyses of developing Aspergillus fumigatus biofilms reveal metabolic shifts required for biofilm maturation
Source: mSphere. 2025 Nov 28;10(12):e00752-25. doi: 10.1128/msphere.00752-25 (PMC12724364; doi:10.1128/msphere.00752-25)
Supplement: Fig. S6 — Acetoin accumulation in culture supernatants of low oxygen cultures. [file msphere.00752-25-s0006.pdf]

Figure S6

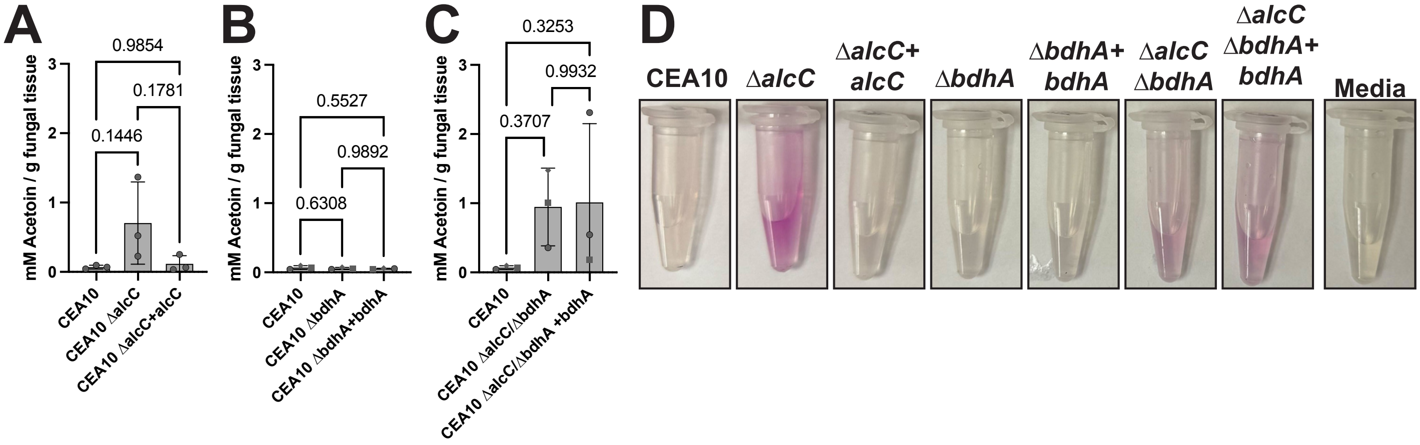

Figure S6. Acetoin accumulation in culture supernatants of low oxygen cultures. Acetoin levels were quantified in culture supernatants from shaking cultures that were grown for 24 hours in atmospheric oxygen supplemented with 5% CO2 switched into 0.2% O2 supplemented with 5% CO2 conditions for 48 additional hours. **A**) Quantification of acetoin in culture supernatants using a Voges-Proskauer assay. **B**) representative images of colorimetric output from acetoin detection analysis. Statistics are a one-way ANOVA with a Tukey's multiple comparison test.
