# Supplementary material for: Transcriptional and metabolic modeling analyses of developing Aspergillus fumigatus biofilms reveal metabolic shifts required for biofilm maturation
Source: mSphere. 2025 Nov 28;10(12):e00752-25. doi: 10.1128/msphere.00752-25 (PMC12724364; doi:10.1128/msphere.00752-25)
Supplement: Fig. S7 — Heatmap of kinase transcript abundance with hierarchical clustering. [file msphere.00752-25-s0007.pdf]

# Figure S7

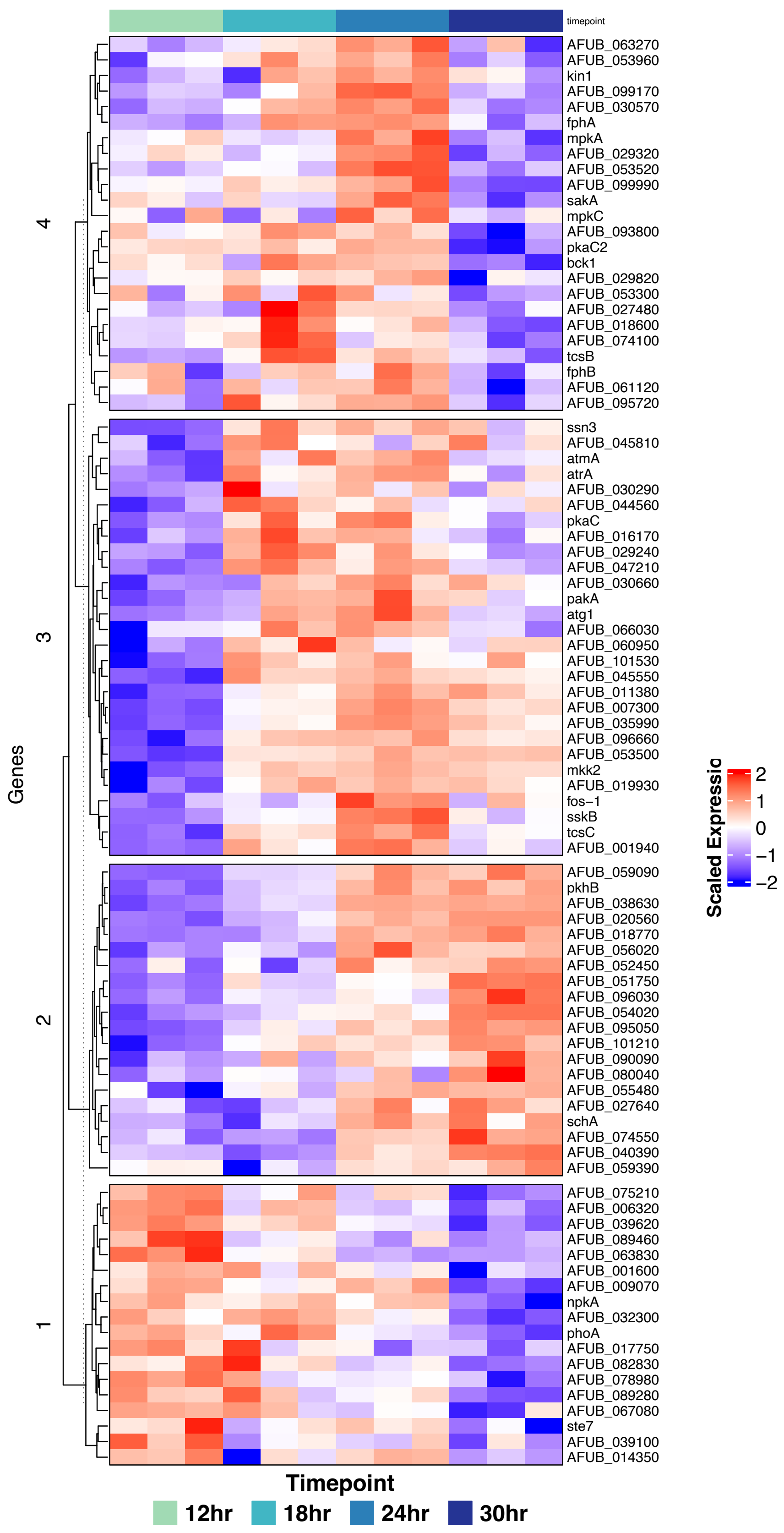


**Figure S7**: Heatmap of kinase transcript abundance with hierarchical clustering. Scaled CPM values are shown. K-means cluster number is indicated to the left of the heatmap.
