# Supplementary material for: Transcriptional and metabolic modeling analyses of developing Aspergillus fumigatus biofilms reveal metabolic shifts required for biofilm maturation
Source: mSphere. 2025 Nov 28;10(12):e00752-25. doi: 10.1128/msphere.00752-25 (PMC12724364; doi:10.1128/msphere.00752-25)
Supplement: Fig. S8 — AFUB_013240 is dispensable for colony biofilm growth in hypoxia. [file msphere.00752-25-s0008.pdf]

Figure S8

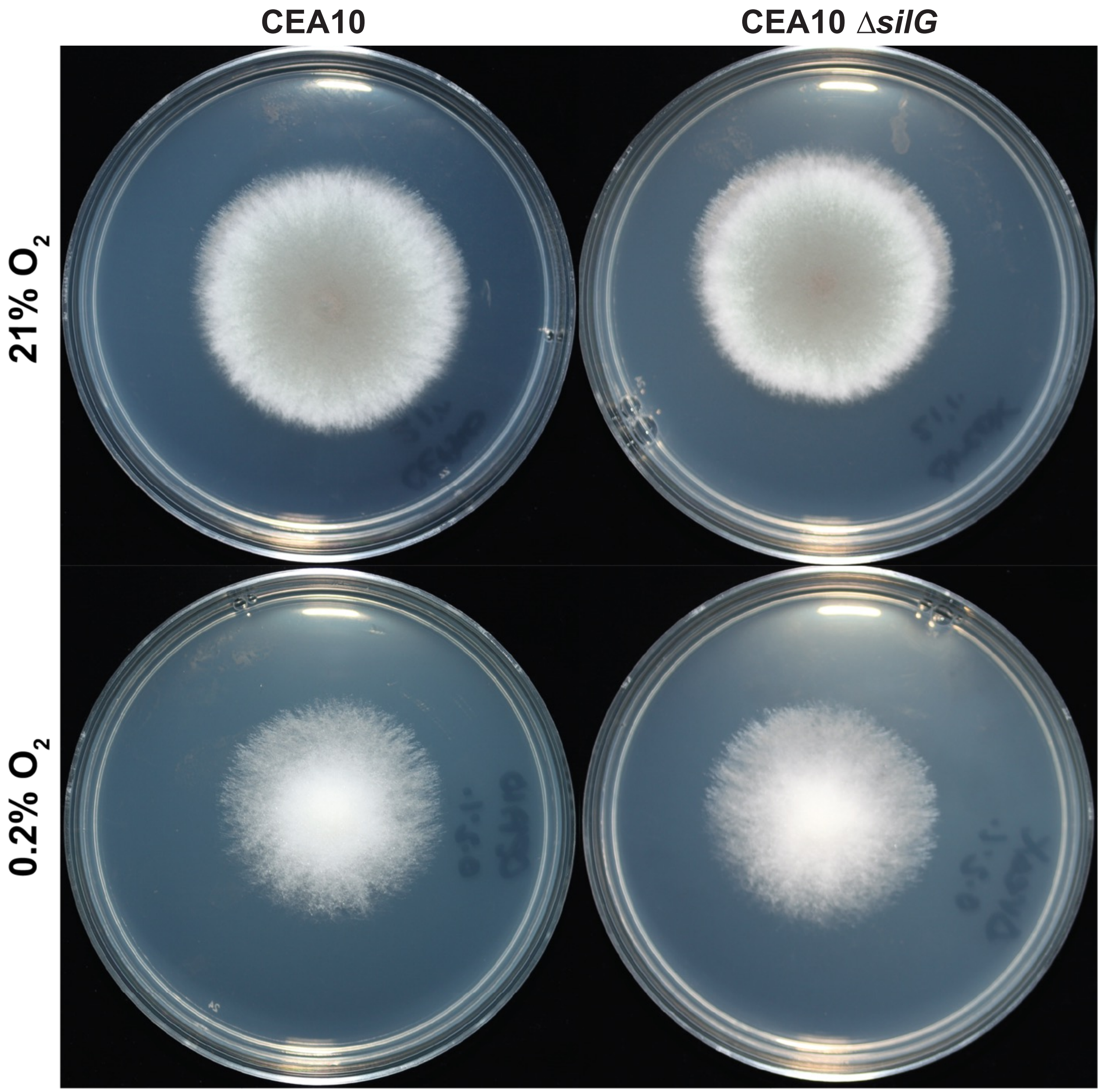

Figure S8: AFUB_013240 is dispensable for colony biofilm growth in hypoxia. Representative images of colony biofilm morphology of ΔAFUB_013240 growth on solid glucose minimal media at 21% and 0.2% O2 compared to wildtype CEA10.
